# Alternative organelle targeting of OPA1 mediates fatty acid release from lipid droplets

**DOI:** 10.64898/2026.05.07.723579

**Published:** 2026-05-11

**Authors:** Xiao Li, Dennis Voronin, Rikshita Bhattacharyya, Jonathon Klein, MaryEllen Haas, Woo Jung Cho, Camenzind G Robinson, Robert E. Throm, Gang Wu, Chang Li, Yadav Sapkota, Natalie M. Niemi, Shondra M. Pruett-Miller, Joseph T. Opferman, Chi-Lun Chang

**Affiliations:** 1 Department of Cell and Molecular Biology, St. Jude Children’s Research Hospital, Memphis, TN, USA; 2 St. Jude Graduate School of Biomedical Sciences, St. Jude Children's Research Hospital, Memphis, TN, USA; 3 Center for Advanced Genome Engineering, St. Jude Children's Research Hospital, Memphis, TN, USA; 4 Department of Physiology, University of Tennessee Health Science Center, Memphis, TN, USA; 5 Cell and Tissue Imaging Center, St. Jude Children’s Research Hospital, Memphis, TN, USA; 6 St. Jude Vector Laboratory, St. Jude Children’s Research Hospital, Memphis, TN, USA; 7 Center for Applied Bioinformatics, St. Jude Children's Research Hospital, Memphis, TN, USA; 8 Department of Pathology, St. Jude Children's Research Hospital, Memphis, TN, USA; 9 Department of Epidemiology and Cancer Control, St. Jude Children's Research Hospital, Memphis, TN, USA; 10 Department of Biochemistry and Molecular Biophysics, Washington University School of Medicine, St. Louis, MO, USA

## Abstract

Mitochondria and lipid droplets (LDs) are functionally coupled to coordinate fatty acid utilization and storage. However, a comprehensive understanding of mitochondria–LD alliances remains elusive. We have identified a previously unrecognized role for optical atrophy 1 (OPA1), a mitochondrial fusion factor, in the regulation of fatty acid release from LDs. We demonstrated that OPA1’s exon 4 adapts an amphipathic helix to target OPA1 to LDs. OPA1 localized to LDs promote fatty acid release by facilitating the recruitment of lipases to LDs. In addition, OPA1’s residence on LDs competes with its mitochondrial entry, influencing mitochondria fusion and connectivity. Furthermore, the S158N polymorphism within OPA1’s exon 4 exhibiting attenuated fatty acid release from LDs is associated with changes in metabolic traits in pediatric cancer survivors. Altogether, our findings reveal that OPA1 actively mediates fatty acid release from LDs and provide a mechanistic link between OPA1 and human metabolism.

Mitochondria are the central hub for energy homeostasis, integrating products from the glycolysis-pyruvate pathway and fatty acid oxidation (FAO) into the tricarboxylic acid (TCA) cycle to generate ATP and support energetic adaptations. Unlike pyruvate, which is readily available in the cytoplasm, fatty acids (FAs) are stored within lipid droplets (LDs) as triacylglycerols (TAGs) and cholesterol-esters (CEs)^1, 2^, and must be released from the LDs prior to mitochondrial import for FAO and ATP production. The release of FAs from LDs is mediated through lipolysis via the coordinated actions of lipases^3, 4^. TAGs, for example, are hydrolyzed sequentially by the adipose triglyceride lipase (ATGL), hormone-sensitive lipase (HSL), and monoacylglycerol lipase (MAGL) on the surface of LDs and become free FAs and glycerol. The recruitment of lipases to LDs is central to lipolysis and regulated by protein kinase A (PKA) during active lipolytic signaling^5-8^. Additionally, ATGL and HSL, all play essential roles in the TAG cycling, where FAs released through lipolysis are re-esterified into TAGs; this cycling is likely involved in buffering nutrient fluctuations, preventing lipotoxicity, lipid diversification, and thermogenesis^9-12^. While lipolysis and the TAG cycling are well characterized in adipose tissue, increasing evidence suggests these pathways exist across diverse cell types^13-15^. However, the molecular mechanisms governing lipase recruitment to and activation on LDs in non-adipose cell types remain poorly defined.

Cytosolic coenzyme A (CoA)-conjugated FAs can be imported into mitochondria, diverting them from storage, leading to a net loss of FA from LDs. Emerging evidence suggests that mitochondrial morphology and connectivity impact mitochondrial FA import and oxidation. Genetic perturbation promoting mitochondrial fragmentation in fibroblasts resulted in lipid droplet accumulation, a phenotype thought to arise indirectly from compromised mitochondria FA uptake and FAO^16, 17^. Similarly, induced mitochondria elongation in mouse adipocytes was associated with enhanced FAO and reduced TAG storage^18, 19^. However, in hepatocytes, pancreatic islet β-cells, and certain lymphomas, mitochondria that have been fragmented by nutrient or genetic perturbations were found to favor FAO *in vitro*^20^. In metastatic breast cancer cells, increased FAO was accompanied by enhanced mitochondrial fission^21^. Additionally, LD-associated mitochondria with elongated morphology isolated from mouse brown adipose tissues had reduced capacity for FAO and favored TAG synthesis, leading to the expansion of LDs^22^. These apparently contradictory findings highlight a key unresolved question: how does mitochondrial morphology mechanistically influence dynamic FA storage within LDs? Notably, most existing studies have examined LD abundance only as a secondary outcome of altered mitochondrial dynamics without directly interrogating the capability of LD biogenesis and breakdown. Even though the functional coordination between LDs and mitochondria is crucial for sustaining cellular homeostasis, it is not well understood how mitochondria coordinate FA release from LDs.

One such ‘missing’ coordinator may be optical atrophy 1 (OPA1); OPA1 is a well characterized and broadly expressed mitochondrial morphological factor that promotes inner mitochondrial membrane fusion via its GTPase activity^23^. In humans, eight OPA1 mRNA isoforms are produced by alternative splicing of exons 4, 4b, and 5b^24^. Following translation and mitochondrial import, OPA1 is anchored on the inner membrane as the long OPA1 (L-OPA1) isoforms, which are subjected to further proteolytic processing to generate soluble, short OPA1 (S-OPA1) isoforms in the intermembrane space^25^. Whether inner membrane fusion can be mediated by L-OPA1 alone, or instead requires both L-OPA1 and S-OPA1, remains an open question^26-30^. In addition to its roles within mitochondria, OPA1 appeared to have non-canonical functions in regulating lipid storage. For example, OPA1 has been detected on LDs in cultured adipocytes and adrenal gland cells; however, whether it plays a role in PKA-mediated signaling appears to be cell-type dependent^31, 32^. Multiple proteomic studies have also identified OPA1 in the LD proteome in mouse skeletal muscle, cultured hepatocytes, and U2OS and HEK293 cell lines^33-35^. In mice, OPA1 promotes white adipose tissue expansion and browning^36, 37^ and is required for lipid metabolism and cold-induced thermogenic activation^37, 38^. Despite these findings, the precise molecular mechanisms by which OPA1 orchestrates LD remodeling, and whether these mechanisms extend to non-adipose cells, are unknown.

Here, we report that OPA1 mediates FA release via exon 4-directed LD targeting. Through comprehensive analyses of FA fluxes, we demonstrate that, in addition to canonical functions in mitochondria, OPA1 actively regulates FA release from LDs via two interrelated mechanisms: (1) first, exon 4 adopts an amphipathic helix that dynamically directs a subpopulation of OPA1 to LDs. A naturally occurring OPA1 isoform 2, which lacks exon 4, is exclusively distributed to mitochondria. (2) Second, LD-associated OPA1 facilitates the recruitment of ATGL and HSL to LDs under resting conditions. The recruitment of these lipases and the consequent FA release positively correlate with the level of OPA1 on LDs. Additionally, OPA1’s residence on LDs effectively reduces its mitochondrial localization and therefore reduces mitochondria fusion and connectivity. Notably, a common human polymorphism (*i.e.,* S158N within exon 4) decreases OPA1 targeting to LDs and is associated with elevated body fat and other metabolic traits from a database of cancer survivors. Altogether, our findings reveal a new role for the ubiquitously expressed OPA1 in mediating FA release from LDs and suggest a mechanistic link between LD metabolism and mitochondrial morphology through OPA1’s alternative organelle targeting.

## Results

### Defective FA release from LDs in OPA1 knockout cells

**Figure 1.**
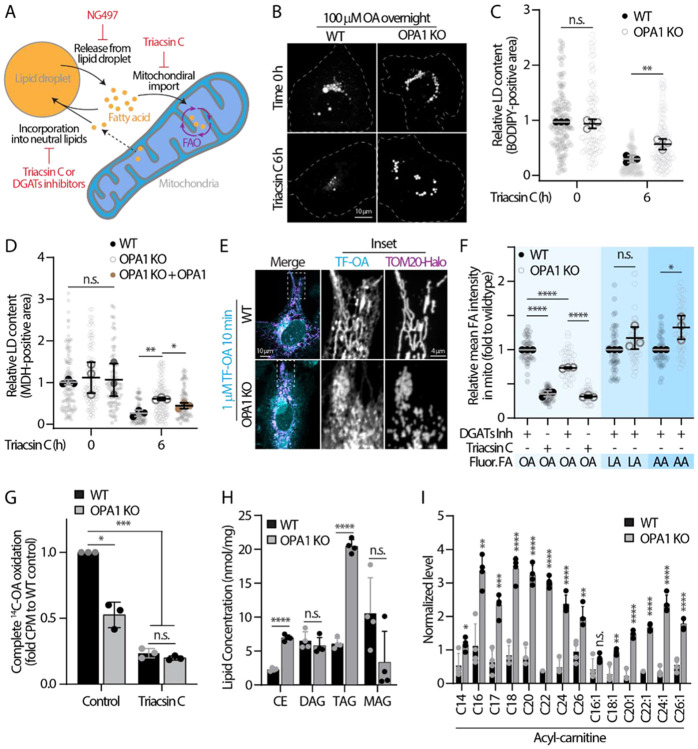
Comprehensive analysis of fatty acid trafficking reveals defective fatty acid release from lipid droplets in *OPA1* knockout cells. **(A)** Multidirectional fatty acid (FA) trafficking pathways between lipid droplets (LDs) and mitochondria. Terms: FAO, fatty acid oxidation; NG497, an ATGL inhibitor; Triacsin C, an inhibitor of long FA acyl-CoA synthetase. **(B)** BODIPY 493/503-labeled LDs detected via confocal microscopy before and after 6-h incubation with 10 μM Triacsin C in wildtype (WT) and *OPA1* knockout (KO) U2OS cells treated with 100 μM oleic acid (OA) overnight. Maximal intensity projected (MIP) confocal images from whole cells are shown. Dashed lines mark cell boundary. **(C)** Quantification of BODIPY 493/503-positive LD content as described in (B). Mean ± standard deviation from three independent experiments are shown (total of 99–161 cells). n.s., no significance, **P ≤ 0.001, as assessed by one-way ANOVA. **(D)** Quantification of monodansylpentane (MDH)-positive LD content in WT, *OPA1* KO, and OPA1-expressing *OPA1* KO U2OS cells treated with 100 μM OA overnight followed by 10 μM Triacsin C incubation for 6 h. Mean ± standard deviation from three independent experiments are shown (total of 81–115 cells). n.s., no significance, **P ≤ 0.01, *P ≤ 0.05, as assessed by one-way ANOVA. **(E)** Subcellular localization of TopFluor (TF)-OA and TOM20-Halo (JF646) detected via confocal microscopy in WT and OPA1 KO U2OS cells treated with 20 μM DGAT1 and DGAT2 inhibitors. MIP confocal images from three axial slices (~0.6 μm total thickness) are shown. **(F)** Quantification of the intensity of peripheral mitochondria (mito) of TF-OA as described in (E) and in cells pretreated with 10 μM Triacsin C, 1 μM BODIPY-linoleic acid (LA), or 1 μM NBD-arachidonic acid (AA). Mean ± standard deviation from three independent experiments are shown (total of 38–50 cells). n.s., no significance, ****P ≤ 0.0001, *P ≤ 0.05, as assessed by one-way ANOVA. **(G)** Scintillation counts per minute (CPM) for complete ^14^C-OA oxidation in WT and *OPA1* KO U2OS cells under control and Triacsin C-treated conditions. Mean ± standard deviation from three independent experiments are shown. n.s., no significance, ***P ≤ 0.001, *P ≤ 0.05, as assessed by one-way ANOVA. **(H)** Concentration of cholesterol-ester (CE), diacylglycerol (DAG), triglyceride (TAG), and monoacylglycerol (MAG) in steady-state WT and *OPA1* KO U2OS cells determined via liquid chromatography–mass spectrometry. Mean ± standard deviation from four replicates are shown. n.s., no significance, ****P ≤ 0.0001, as assessed by unpaired *t*-test. **(I)** Variance in acyl-carnitine levels across acyl-chain length in steady-state WT and *OPA1* KO U2OS cells determined using liquid chromatography–mass spectrometry. Mean ± standard deviation from four replicates are shown. n.s., no significance, ****P ≤ 0.0001, ***P ≤ 0.001, **P ≤ 0.01, *P ≤ 0.05, as assessed by unpaired *t*-test.

LDs and mitochondria are functionally coupled to support FA metabolism (Fig. 1A). To investigate how OPA1 impacts LDs, we used a battery of assays to selectively characterize FA release from LDs, mitochondrial FA import, and mitochondrial FAO in wildtype (WT) and *OPA1* knockout (KO) U2OS cells generated via CRISPR-genome editing and validated by sequencing, western blotting, and immunostaining (Fig. S1A-C). We first asked if FA release from LDs is affected by OPA1 by subjecting cells to oleic acid (OA) withdrawal in the presence of Triacsin C, an inhibitor of long FA acyl-CoA synthases, to blunt mitochondrial FA import and LD biogenesis simultaneously, promoting net FA release from LDs^39-42^. WT cells were first treated with 100 μM OA overnight to induce LD accumulation; followed by a 6-h incubation with Triacsin C, we observed a substantial reduction (23% remaining) in LD content (Fig. 1B, C). We confirmed the Triacsin C-mediated reduction was indeed FA release from LDs because Triacsin C co-incubation with NG497, an ATGL inhibitor^43^, but not co-incubation with Lalistat 2, a lysosomal lipase inhibitor, prevented the loss of LDs (Fig. S2A). Co-incubation of Triacsin C and H89, a PKA inhibitor^44^, minimally affected LD breakdown (Fig. S2B), suggesting that PKA did not participate in the Triacsin C-mediated FA release in resting state. Intriguingly, when comparing *OPA1* KO U2OS and WT cells with similar baseline LD content following OA incubation overnight, the KO cells displayed significantly higher LD accumulation (59% remaining) than WT cells following Triacsin C treatment (Fig. 1B, C), suggesting defects in FA release from LDs. This defective FA release was also observed in *OPA1* knockdown HeLa cells and *OPA1* KO mouse embryonic fibroblasts (MEFs) (Figs. S1D, E and S2C, D), indicating OPA1’s role in regulating FA release may be generalizable across different cell types. Consistent with these observations, we found *OPA1* KO U2OS cells retained significantly higher amounts of LDs than WT cells (60% in KO vs 34% in WT) following glucose starvation for 20 h (Fig. S2E). To continue this characterization, we introduced a yellow fluorescence protein (YFP)-tagged OPA1 isoform 1 (OPA1-YFP), the most abundant OPA1 isoform, into the *OPA1* KO cells via lentiviral transduction and found that the accumulated LD content was significantly reduced after the 6-h Triacsin C incubation (Fig. 1D), suggesting that OPA1 plays an active role mediating FA release. Furthermore, we assessed the levels of key enzymes (*e.g.,* DGAT1, ATGL, HSL and CGI-58) involved in LD biogenesis and breakdown and found the observed FA release defect in *OPA1* KO cells was not due to compensatory mechanisms associated with aberrant expressions of these key enzymes (Fig. S1B, F).

We next sought to directly assess mitochondrial FA import. First, we transiently pulsed cells with 1 μM fluorescently labeled FAs for 10 min in the presence of DGAT1 and DGAT2 inhibitors to eliminate FA incorporation into LDs, as this incorporation competes with mitochondrial import. Following the transient pulse, TopFluor (TF)-OA signal was readily detected in mitochondria in both WT and *OPA1* KO U2OS cells, regardless of their elongated or fragmented mitochondrial morphology, respectively (Fig. 1E). Quantification of TF-OA intensity in mitochondria showed a 20% reduction of FA import in *OPA1* KO cells compared to WT cells (Fig. 1F). In addition, FAO of ^14^C-labeled OA was greatly reduced in *OPA1* KO cells (Fig. 1G), consistent with data from prior studies^45, 46^. Triacsin C treatment blunted TF-OA uptake and ^14^C-OA FAO in both cell types (Fig. 1F, G), confirming these labeled OA were indeed trafficked into mitochondria. Coincidently, import of BODIPY-linoleic acid (LA) and NBD-arachidonic acid (AA) was, nonetheless, moderately enhanced in *OPA1* KO cells (Fig. 1E). Altogether, these results suggest that *OPA1* KO did not drastically affect overall mitochondrial FA import while it severely limited FAO.

To corroborate these observations, we performed lipidomic and metabolomic analyses in WT and *OPA1* KO U2OS cells in steady state. TAG and CE levels were ~3-fold higher in *OPA1* KO cells than in WT cells (Fig. 1H). *OPA1* KO cells also displayed markedly lower MAG levels than the WT (Fig. 1H); as MAG is a broken-down product from TAG hydrolysis by lipases, this is consistent with the observed reduction in FA release from LDs in *OPA1* KO cells. Additionally, overall lipid synthesis was not as strongly affected in the *OPA1* KO cells, though several phospholipid species were moderately upregulated (Fig. S2F, G). Furthermore, nearly all detected acyl-carnitine species levels were much higher in *OPA1* KO cells than in WT cells (Fig. 1I), reflecting defective FAO in the OPA1-depleted mitochondria. Altogether, our data demonstrates that OPA1, in addition to its canonical functions in mitochondrial morphology and FAO, can directly impact FA release from neutral lipids within LDs in the absence of lipolytic signaling.

### A subpopulation of OPA1 localizes to LDs

**Figure 2.**
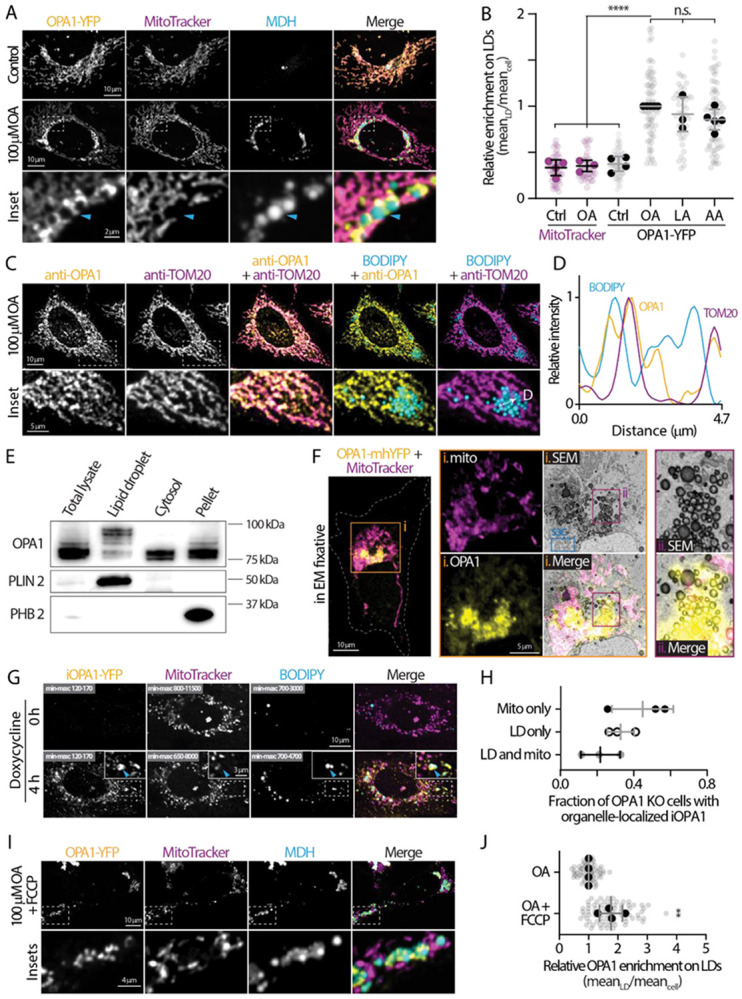
A subpopulation of OPA1 is localized to lipid droplets. **(A)** Localization of OPA1-YFP (lentiviral), mitochondria (labeled with MitoTracker Deep Red), and lipid droplets (LDs; labeled with MDH) in U2OS cells −/+ overnight 100 μM oleic acid (OA) treatment. Maximal intensity projected (MIP) confocal images from four axial slices (~1 μm total thickness) are shown. Cyan arrowheads indicate area with LDs. **(B)** Relative enrichment of MitoTracker and OPA1 on LDs from (A) and in cells treated with 100 μM linoleic acid (LA) or arachidonic acid (AA) overnight. Mean ± standard deviation from 3–5 independent experiments are shown (total of 43–74 cells). n.s., no significance, ****P ≤ 0.0001, assessed by one-way ANOVA. **(C)** Subcellular localization of endogenous OPA1 on mitochondria (anti-TOM20) and LDs labeled by BODIPY 493/503 in an OA-treated U2OS cell monitored with confocal microscopy. Sum of confocal images from five axial slices (~1.2 μm in total thickness) are shown. **(D)** Relative intensity profiles of OPA1, TOM20, and BODIPY measured along the white-dashed arrow from lower left panel in (C). **(E)** Western blot analysis of endogenous OPA1, PLIN 2 (an LD protein), and PHB 2 (a mitochondrial inner membrane protein) in sucrose-gradient cellular fractionations from U2OS cells treated with 500 μM OA. **(F)** Correlative confocal-scanning electron microscopy (SEM) images of OPA1 on LDs and in mitochondria in OPA1-mhYFP–expressing U2OS cells treated with 100 μM OA overnight. Confocal and SEM images from a single axial slice are shown. EM, electron microscopy. Inset outlined in blue is shown in Fig. S3C. **(G)** Subcellular distribution of inducible OPA1 (iOPA1)-YFP on LDs (BODIPY 665/676) and in mitochondria (MitoTracker Red) in *OPA1* KO U2OS cells following treatment with 4 μg/mL doxycycline for 4 h. Confocal images from a single axial slice are shown. Cyan arrowheads indicate regions containing LDs. **(H)** Fraction of cells with iOPA1 localized to indicated organelle as described in (G). Mean ± standard deviation from three independent experiments are shown. **(I)** Localization of OPA1-YFP, mitochondria (MitoTracker Deep Red), LDs (MDH) in U2OS cells treated with 100 μM OA and 20 μM FCCP (an uncoupler of mitochondria oxidative phosphorylation) overnight detected by confocal microscopy. MIP confocal images from four axial slices (~1 μm total thickness) are shown. **(J)** Relative enrichment of OPA1 on LDs in U2OS cells treated with 100 μM OA −/+ 20 μM FCCP for overnight in (I). Mean ± standard deviation from four independent experiments are shown (total of 57–73 cells). **P ≤ 0.01, as assessed by unpaired *t*-test.

To understand how OPA1 residing within mitochondria can play a role in releasing FA from LDs, we examined the localization of OPA1-YFP in in resting and LD-laden U2OS cells. While OPA1 exclusively colocalized with mitochondria in resting cells as detected by confocal microscopy, a subpopulation of OPA1-YFP resided on LDs when cells were treated with OA overnight (Fig. 2A). This shift in distribution is quantified as relative OPA1 enrichment on LDs and this shift was also observed in WT U2OS cells treated with LA or AA to induce LD formation (Figs. 2B and S3A), suggesting that the FA content within LDs is not a crucial determinant for OPA1’s localization. Consistent with this observation, a subpopulation of OPA1-YFP was also found on LDs in HeLa and Huh7 cells treated with OA overnight (Fig. S3B). In addition, a small population of endogenous OPA1 was observed on clusters of small LDs in U2OS cells via immunostaining and line scanning analysis using an OPA1 antibody validated in *OPA1* KO U2OS cells (Figs. 2C, D, and S1C). Subcellular fractionation via ultracentrifugation further revealed that the endogenous L-OPA1 (top high-molecular weight bands) were enriched in the LD fraction while the S-OPA1 (lower bands) generated via mitochondrial proteolysis were primarily present in the cytosol and pellet (total membrane) fractions (Fig. 2E). We next performed correlative confocal-scanning electron microscopy (SEM) to validate these observations with high spatial resolution. We expressed a monomeric hyperfolder-YFP tagged OPA1 (OPA1-mhYFP) that retains fluorescence in EM grade fixatives^47^ in U2OS cells stained with MitoTracker as a fiducial marker for correlative SEM. In addition to regions enriched with mitochondria (Fig. S3C), OPA1-mhYFP signals were found in areas containing small LDs without the presence of mitochondria (Fig. 2F), demonstrating that OPA1 indeed resides on the surface of LDs.

OPA1 is a nuclear-encoded protein that was not detected in previous co-translationally inserted mitochondrial proteomes^48, 49^, suggesting it may be translated in the cytosol prior to reaching its organelle destination. To interrogate the timing of OPA1’s targeting of LDs following its translation, we introduced an inducible OPA1-YFP (iOPA1-YFP) under a tetracycline-driven promoter into *OPA1* KO U2OS cells to avoid endogenous OPA1-directed distribution via dimerization. The iOPA1-YFP signals were detected on both mitochondria and LDs after ~4 h of doxycycline induction (Fig. 2G). Although the signal was dim, we were able to categorize the iOPA1-YFP signal based on its first detected organelle distribution and found that 45% of the cell population has this signal exclusively in mitochondria, 33% has it exclusively on lipid droplets, and 22% showed the signal in both organelles (Fig. 2H). We next applied carbonyl cyanide-p-trifluoromethoxyphenylhydrazone (FCCP) to disrupt mitochondrial protein import by collapsing the proton gradient^50^ and found that OPA1-YFP was primarily distributed to LDs (Fig. 2I, J). In addition, endogenous OPA1 was more readily detected on clustered LDs after FCCP treatment (Fig. S3D, E vs Fig. 2C, D).

Dual localization of mitochondrial proteins has been linked to a ‘weak’ mitochondrial targeting sequence (MTS) which disallows efficient mitochondrial import to promote alternative subcellular localization^51, 52^. We wondered if OPA1 may have a weak MTS facilitating its localization at LDs. Using OPA1’s previously mapped MTS (87 amino acids in length^28^), we calculated its amphiphilicity, a product of the charge and maximal hydrophobic moment of a MTS, and found it had the highest score compared with a dozen of experimentally determined MTSs across multiple species (Figures S3F, G), predicting a strong import behavior^53^. These data strongly suggest OPA1’s extramitochondrial localization is not due to a ‘weak’ MTS for inefficient mitochondrial import but rather results from inclusion of a LD-targeting motif required to direct L-OPA1 to LDs shortly following its production in the cytoplasm.

### Exon 4 of OPA1 constitutes an amphipathic helix for LD targeting

**Figure 3.**
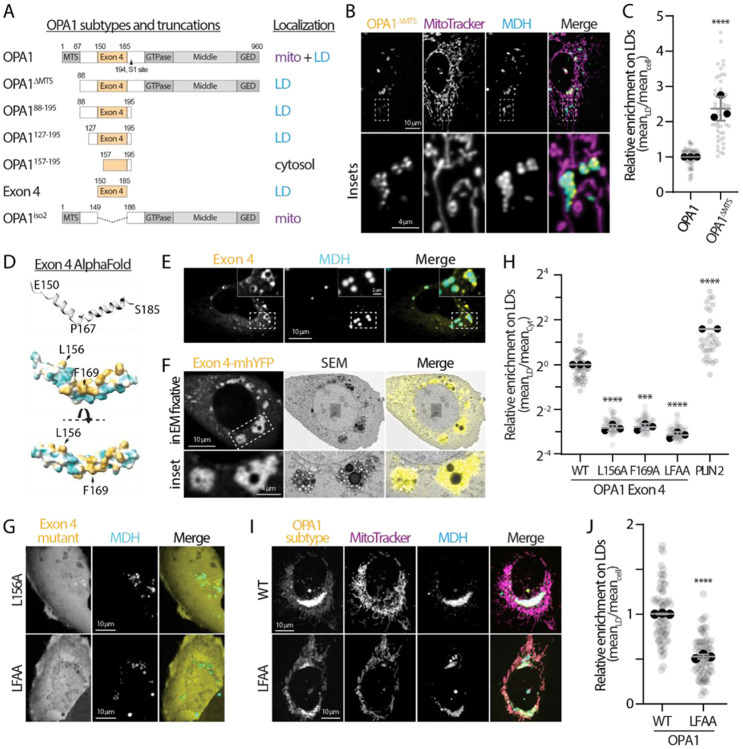
OPA1’s exon 4 adapts an amphipathic helix structure to target lipid droplets. **(A)** Constructs expressing full-length and truncated OPA1, as well as summary of their primary subcellular localizations. Amino acid number and protein domains are indicated. MTS, mitochondria targeting sequence; GED, GTPase effector domain; LD, lipid droplet; mito, mitochondria. **(B)** Localization of OPA1^ΔMTS^-YFP, mitochondria (labeled with MitoTracker Deep Red), and lipid droplets (LDs; labeled with MDH) in U2OS cells treated with 100 μM oleic acid (OA) overnight and detected by confocal microscopy. Maximal intensity projected (MIP) confocal images from four axial slices (~1 μm total thickness) are shown. **(C)** Relative enrichment of OPA1^ΔMTS^ from (B) and OPA1 on LDs in U2OS cells treated with 100 μM OA overnight. Mean ± standard deviation from three independent experiments are shown (total of 48–55 cells). ****P ≤ 0.0001, as assessed by unpaired *t*-test. **(D)** Amphipathic helix structure of exon 4 as predicted by AlphaFold. Cyan and yellow indicate polar and non-polar amino acids, respectively, and black arrows indicate location of residues for mutagenesis. **(E)** Localization of YFP-tagged exon 4 in MDH-stained U2OS cells treated with 100 μM OA overnight and detected by confocal microscopy. MIP confocal images from four axial slices (~1 μm total thickness) are shown. **(F)** Correlative confocal-scanning electron microscopy (SEM) images of exon 4 on LDs in exon 4-mhYFP expressing U2OS cells treated with 100 μM OA overnight. Confocal and SEM images from a single axial slice are shown. **(G)** Localization of exon 4 mutants relative to LDs (MDH) in U2OS cells treated with 100 μM OA overnight and detected by confocal microscopy. MIP confocal images from four axial slices (~1 μm total thickness) are shown. LFAA, a double mutation of L156 and F169 to alanines. **(H)** Relative enrichment of exon 4 and mutants (described in E and G), as well as PLIN 2-GFP on LDs in U2OS cells treated with 100 μM OA overnight. Mean ± standard deviation from two or three independent experiments are shown (total of 30–52 cells). ****P ≤ 0.0001, ***P ≤ 0.001, as assessed by one-way ANOVA. **(I)** Localization of OPA1 and OPA1^LFAA^, mitochondria (MitoTracker Deep Red), and LD (MDH) in U2OS cells treated with 100 μM OA overnight and detected by confocal microscopy. MIP confocal images from four axial slices (~1 μm total thickness) are shown. **(J)** Relative enrichment of OPA1 and OPA1^LFAA^ from (I) on LDs in cells treated with 100 μM OA overnight. Mean ± standard deviation from three independent experiments are shown (total of 63–65 cells). ****P ≤ 0.0001, as assessed by one-way ANOVA.

To identify OPA1’s lipid droplet targeting motif, we generated YFP-tagged, truncated forms of OPA1 focusing on its first 195 amino acids that are exclusively present in L-OPA1 (Fig. 3A)^28^. OPA1 with deleted MTS (OPA1^ΔMTS^) only showed LD distribution (Fig. 3B, C), indicating MTS is not required for LD targeting and an additional LD-targeting motif must reside within amino acids 88–195. While fragments of OPA^88-195^ and OPA1^127-195^ primarily localized to LDs, OPA1^157-195^ with a truncated exon 4 became cytosolic (Figs. 3A and S3H). This observation prompted us to hypothesize that exon 4 is required for OPA1 LD distribution.

AlphaFold 3 predicts that exon 4 adapts a secondary structure of an amphipathic helix with proline^167^ close to the center slightly bending the helix (Fig. 3D). Exon 4 is located within an unstructured region of OPA1 that is readily exposed to the cytoplasm (AlphaFold 3 prediction; data not shown). The non-polar residues (Fig. 3D, yellow) are slightly offset along the long axis, suggesting a weak interaction with membranes. Nonetheless, overexpressed exon 4-YFP displayed clear LD localization in U2OS cells (Fig. 3E). Correlative confocal-SEM further revealed that Exon 4-mhYFP localized to clusters of LDs (Fig. 3F). Mutations of the non-polar amino acids, leucine^156^, phenylalanine^169^, or both, to alanine completely disrupted exon 4’s ability to localize to LDs (Fig. 3G, H). Consistent with this observation, full-length OPA1^LFAA^ also showed significantly weakened accumulation on LDs (Fig. 3I, J), emphasizing the requirement of these non-polar residues for OPA1’s distribution to LDs. Intriguingly, the affinity of exon 4 for LDs was significantly reduced compared to the LD-resident protein, perilipin 2 (PLIN 2) (Fig. 3H), suggesting a more dynamic interaction between LDs and OPA1 via exon 4 coordinates OPA1’s mitochondrial import and canonical functions.

### A naturally occurring OPA1 isoform 2 lacking exon 4 exclusively localizes to mitochondria

**Figure 4.**
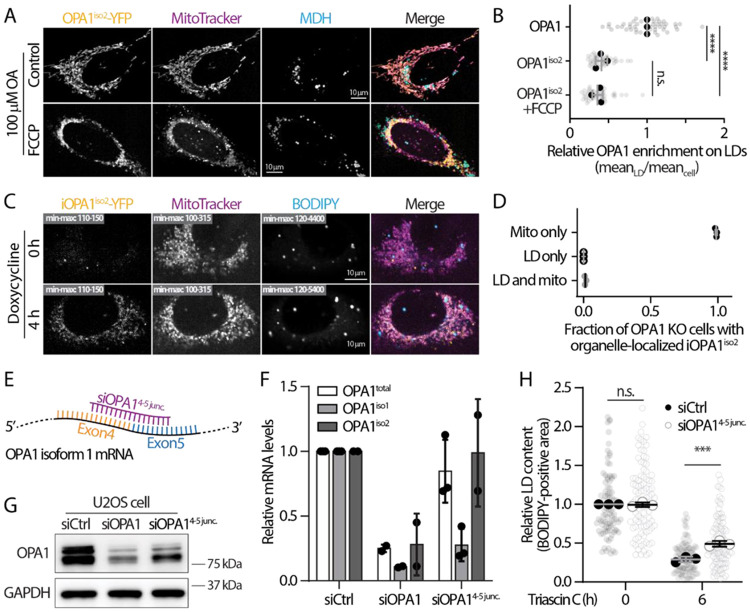
Isoform 2 of OPA1 lacking exon 4 shows minimal localization to lipid droplets. **(A)** Localization of OPA1^iso2^-YFP, mitochondria (labeled with MitoTracker Deep Red), lipid droplets (LDs; labeled with MDH) in U2OS cells treated with 100 μM oleic acid (OA) −/+ 20 μM FCCP (an uncoupler of mitochondria oxidative phosphorylation) overnight detected by confocal microscopy. Maximal intensity projected (MIP) confocal images from four axial slices (~1 μm total thickness) are shown. **(B)** Relative enrichment of OPA1^iso2^ as described in (A) and OPA1 on LDs in U2OS cells treated with 100 μM OA overnight. Mean ± standard deviation from three independent experiments are shown (total of 43–51 cells). n.s., no significance, ****P ≤ 0.0001, as assessed by one-way ANOVA. **(C)** Subcellular distribution of inducible OPA1^iso2^ (iOPA1 ^iso2^)-YFP on LDs (BODIPY 665/676) and mitochondria (MitoTracker Red) in *OPA1* knockout (KO) U2OS cells following 4 μg/mL doxycycline treatment for 4 h. Confocal images from a single axial slice are shown. **(D)** Fraction of cells with iOPA1 ^iso2^ localized to indicated organelle as described in (C). Mean ± standard deviation from three independent experiments are shown. **(E)** Design of an siRNA targeting the OPA1 exon 4–exon 5 junction for selective depletion of isoform 1. **(F and G)** Levels of (F) OPA1 isoform-specific mRNA or (G) protein in U2OS cells transfected with the indicated siRNAs. Mean ± standard deviation from two or three independent experiments are shown in (F). **(H)** Relative BODIPY-positive area indicating LD content in siCtrl and siOPA1^4-5 junc^ transfected U2OS cells treated with 100 μM OA overnight followed by 10 μM Triacsin C incubation for 6 h. Mean ± standard deviation from three independent experiments are shown (total of 90–101 cells). n.s., no significance, ***P ≤ 0.001, as assessed by one-way ANOVA.

Splicing events at exon 4 of OPA1 have been documented in NCBI and UniProt databases. Isoform 2 of OPA1 (OPA1^iso2^) is a variant in which exon 4 is spliced out from the transcript (Fig. 3A). By analyzing the inclusion and exclusion of OPA1 exon 4 from the Genotype-Tissue Expression (GTEx) portal as an indicative expression readout of isoforms 1 and 2, respectively (Fig. S4A), we found that OPA1^iso2^ mRNA is widely expressed in most tissues, albeit at lower levels than OPA1^iso1^ mRNA (Fig. S4A). OPA1^iso2^ transcripts were also expressed in U2OS and HeLa cells (Fig. S4B). Given the importance of exon 4 in OPA1’s LD targeting, we decided to interrogate the subcellular distribution of the naturally occurring OPA1^iso2^. We found YFP-tagged OPA1^iso2^ was only localized in mitochondria in lipid droplet-laden U2OS cells (Fig. 4A), sharply contrasting OPA1 ^iso1^’s LD distribution (Fig. 4A, B). Even under FCCP conditions with compromised mitochondrial import, OPA1^iso2^ remained localized to mitochondria but not on LD in both U2OS and HeLa cells (Figs. 4A, B, and S4C, D). Coincidently, when expressed in *OPA1* KO U2OS cells, inducible OPA1^iso2^ (iOPA1^iso2^-YFP) exclusively entered mitochondria with virtually no cells displaying LD distribution (Fig. 4C, D). These observations further support our hypothesis that exon 4 is required for OPA1’s LD targeting and are consistent with the idea that OPA1 harbors a strong MTS that allows import of OPA1^iso2^ under FCCP treatment.

We next examined whether OPA ^iso1^ alone is required for FA release from LDs using a siRNA targeting the exon 4–exon 5 junction (siOPA1^4-5 junc^) (Figure 4E). U2OS cells transfected with siOPA1^4-5 junc^ showed a specific, substantial reduction of OPA1^iso1^ mRNA accompanied with a prominent depletion of L-OPA1 proteins, while broader depletion of OPA1 mRNA and proteins were observed in siOPA1-transfected cells (Fig. 4F, G). Intriguingly, siOPA1^4-5 junc^-treated U2OS cells displayed significantly more accumulated LDs (49% remaining) than siControl (siCtrl)-transfected cells (29% remaining) after Triacsin C treatment (Fig. 4H), which phenocopied the FA release defect in *OPA1* KO U2OS cells (Fig. 1C). A similar difference in LD content between siCtrl and siOPA1^4-5 junc^ HeLa cells (34% vs 51% remaining) was also observed (Fig. S4E). These results suggest that OPA1^iso1^ is required for FA release from LDs, likely through its ability to reside on LDs.

### LD-localized OPA1 facilitates recruitment of lipases

**Figure 5.**
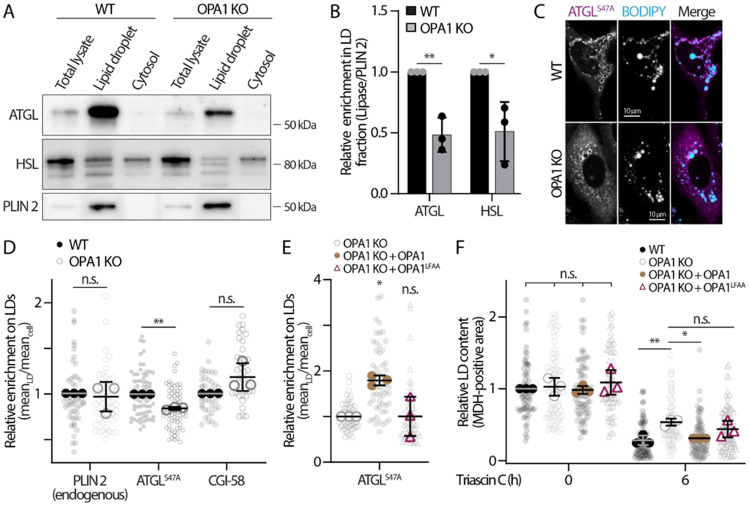
Lipid droplet–associated Opa1 facilitates lipase recruitment. **(A)** Western blot analysis of endogenous ATGL, HSL, and PLIN 2 in sucrose-gradient cellular fractionations from wildtype (WT) and *OPA1* knockout (KO) U2OS cells treated with 500 μM oleic acid (OA). **(B)** ATGL and HSL abundance in lipid droplet (LD) fractions normalized to PLIN2 as described in (A). Mean ± standard deviation from three independent experiments are shown. **P ≤ 0.01, *P ≤ 0.05, as assessed by unpaired *t*-test. **(C)** Localization of ATGL S47A-Halo and LDs (labeled with BODIPY-493/503) in WT and *OPA1* KO U2OS cells treated with 100 μM OA overnight. Maximal intensity projected (MIP) confocal images from four axial slices (~1 μm total thickness) are shown. **(D)** Relative enrichment of endogenous PLIN 2 (detected by immunostaining), ATGL^S47A^-Halo, and mApple-CGI-58 on LDs from Figures S5A, 5C, and S5B, respectively. Mean ± standard deviation from three independent experiments are shown (total of 72–74 cells for ATGL^S47A^-Halo; total of 50-52 cells for PLIN 2, and total of 34–46 cells for mApple-CGI-58). n.s., no significance, **P ≤ 0.01, as assessed by unpaired *t*-test. **(E)** Relative enrichment of ATGL^S47A^-Halo on LDs in *OPA1* KO U2OS cells and *OPA1* KO cells reconstituted with lenti-OPA1-YFP or lenti-OPA1^LFAA^-YFP. Mean ± standard deviation from three independent experiments are shown (total of 56–60 cells). For statistics in panels E and F, n.s., no significance, **P ≤ 0.01, *P ≤ 0.05, as assessed by one-way ANOVA. **(F)** MDH-positive LD content in WT and *OPA1* KO U2OS cells, as well as *OPA1* KO cells reconstituted with lenti-OPA1-YFP or lenti-OPA1^LFAA^-YFP. Cells were treated with 100 μM OA overnight followed by 6-h incubation with 10 μM Triacsin C. Mean ± standard deviation from three independent experiments are shown (total of 71–97 cells).

To gain further insights into how OPA1 mediates FA release from LDs, we interrogated whether OPA1 could facilitate lipases’ access to LDs, which is essential to lipase functions^3, 4^. Subcellular fractionation revealed that ATGL and HSL were significantly enriched in the LD fraction from WT U2OS cells compared to the *OPA1* KO U2OS cells (Figs. 5A, B). Nonetheless, enrichment of endogenous PLIN2 on LDs was comparable in WT and *OPA1* KO U2OS cells when examined by fractionation and immunostaining (Figs. 5A, D, and S5A), eliminating the possibility of a global reduction of the LD proteome in *OPA1* KO cells. Consistent with endogenous ATGL, the enrichment of exogenous ATGL^S47A^-Halo, a catalytically dead ATGL used to assess localization without consuming LDs, was also significantly reduced in *OPA1* KO compared to WT cells (Fig. 5C, D), while overexpressed mApple-CGI-58 displayed comparable distribution to LDs in both cell types (Figs. 5D and S5B). Reconstituting OPA1-YFP, but not OPA1^LFAA^-YFP with reduced LD enrichment, reversed the defects in ATGL^S47A^-Halo enrichment on LDs in *OPA1* KO U2OS cells (Fig. 5E). Similarly, OPA1-YFP restored defective FA release following Triacsin C treatment in *OPA1* KO cells whereas OPA1^LFAA^-YFP had minimal effects (Fig. 5F). Altogether, these findings indicate that OPA1 abundance on LDs is crucial for enriching ATGL and HSL to release FAs from LDs. Given that the cellular expression and LD enrichment of CGI-58 was not changed due to OPA1 depletion (Figs. S1E, S5B, and 5D), we propose that OPA1-mediated lipase recruitment to LDs is independent of canonical lipolytic signaling.

### OPA1’s subcellular distribution differentially regulates mitochondrial characteristics

**Figure. 6:**
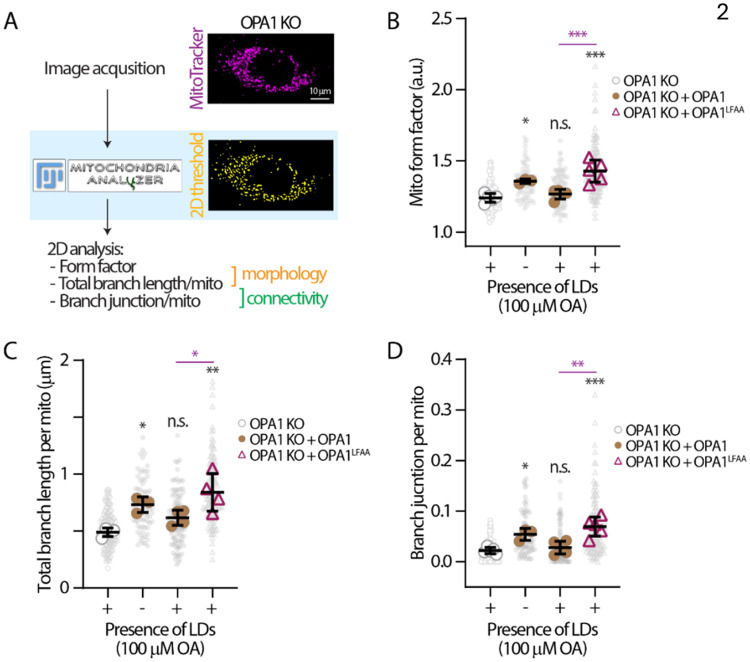
Differential regulation of mitochondrial characteristics depending on OPA1’s subcellular distribution. **(A)** A schematic illustrating two-dimensional (2D) analysis of mitochondrial morphology and connectivity using the Fiji plugin, ‘Mitochondria-Analyzer’. Representative MitoTracker DeepRed images (maximal intensity projected confocal images from three axial slices; 0.6 μm in thickness) from *OPA1* knockout (KO) U2OS cells and the corresponding analyses are shown. **(B–D)** Two-dimensional analysis of mitochondrial morphology and connectivity measuring (B) form factor, (C) total branch length, and (D) branch junction in MitoTracker DeepRed-stained *OPA1* KO U2OS cells and *OPA1* KO U2OS cells reconstituted with lenti-OPA1-YFP or lenti-OPA1^LFAA^-YFP. Mean ± standard deviation from three or four independent experiments are shown (total of 80–133 cells). n.s., no significance, ***P ≤ 0.001, **P ≤ 0.01, *P ≤ 0.05, as assessed by one-way ANOVA. Black asterisks represent comparisons with *OPA1* KO, and purple asterisks represent the indicated comparison.

In addition to facilitating FA release, OPA1’s LD distribution competes with its mitochondrial targeting, likely reducing OPA1’s ability to enter mitochondria and affecting mitochondrial fusion. To test this idea, we applied Mitochondria-Analyzer^54^, a Fiji plugin (Fig. 6A), to first establish baseline 2D measurements of mitochondrial form factor, total branch length, and branch junctions as characteristics of morphology and connectivity in WT and *OPA1* KO U2OS cells. Consistent with prior studies^23, 55, 56^, depletion of OPA1 significantly abolished all mitochondrial characteristics (Fig. S5C-E). While expressing exogenous OPA1-YFP in *OPA1* KO cells partially rescued mitochondrial morphology and connectivity in steady state (Fig. 6B-D), OPA1-YFP failed to rescue mitochondrial characteristics in LD–laden *OPA1* KO cells, a condition under which effective mitochondrial entry of OPA1-YFP is reduced due to its LD distribution. Intriguingly, OPA1^LFAA^-YFP, with a reduced affinity for LDs, restored mitochondrial morphology and connectivity in OA-treated *OPA1* KO cells to levels similar to levels observed in OPA1-YFP-expressing *OPA1* KO cells in steady state. These results support the idea that interaction of OPA1 and lipid droplets can dynamically regulate mitochondrial characteristics via controlling effective OPA1 entry into mitochondria.

### Effects of OPA1 S158N polymorphism on FA release, mitochondrial characteristics, and human metabolic traits

**Fig. 7.**
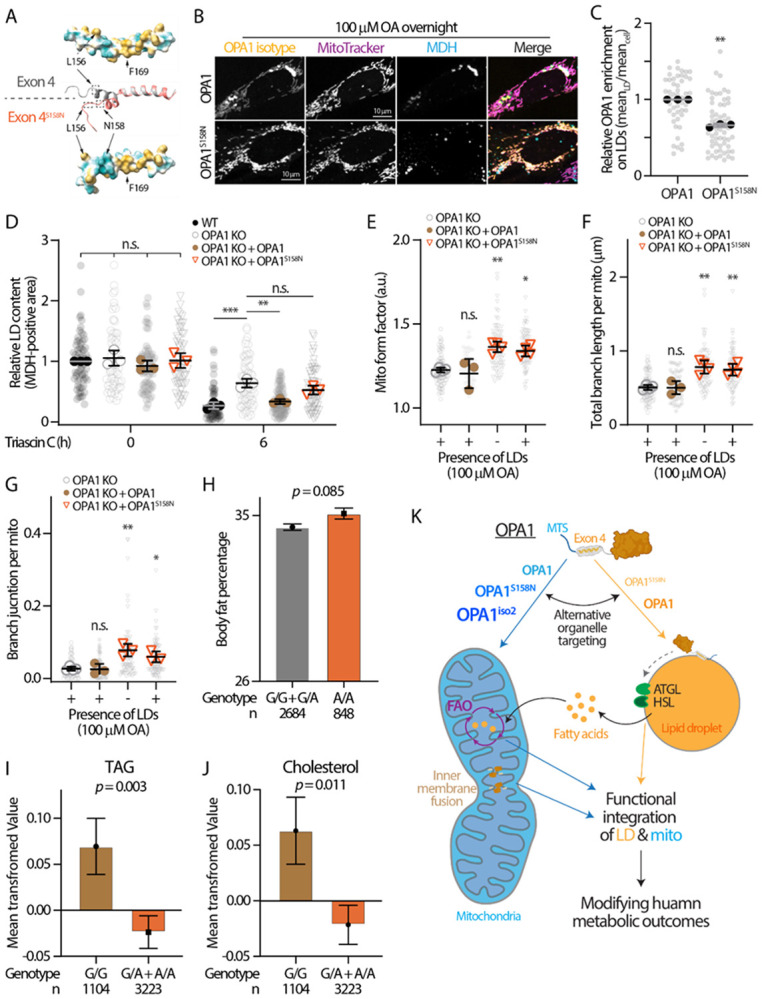
The S158N polymorphism within exon 4 impairs Opa1 targeting to lipid droplets and is associated with clinical manifestations in patients. **(A)** Amphipathic helix structures and hydrophobicity distributions of exon 4 and exon 4 S158N, predicted by AlphaFold and illustrated in ChimeraX. Cyan and yellow indicate polar and non-polar amino acids, respectively, and arrows denote locations of indicated residues. **(B)** Localization of YFP tagged OPA1 or OPA1^S158N^ (lentiviral), mitochondria (labeled with MitoTracker Deep Red), and lipid droplets (LDs; labeled with MDH) in U2OS cells treat with 100 μM oleic acid (OA) overnight. Maximal intensity projected (MIP) confocal images from four axial slices (~1 μm total thickness) are shown. **(C)** Relative enrichment of OPA1 on LDs in U2OS cells as described in (B). Mean ± standard deviation from three independent experiments are shown (total of 50-60 cells). **P ≤ 0.01, assessed by unpaired *t*-test. **(D)** MDH-positive LD content in wildtype (WT) and *OPA1* KO U2OS cells, and in *OPA1* KO cells reconstituted with lenti-OPA1-YFP or lenti-OPA1^S158N^-YFP. Cells were treated with 100 μM OA overnight followed by incubation with 10 μM Triacsin C 6 h. Mean ± standard deviation from three independent experiments are shown (total of 63-83 cells). **(E–G)** Two-dimensional analysis of mitochondrial morphology and connectivity via measuring (E) form factor, (F) total branch length, and (G) branch junction in MitoTracker DeepRed-stained *OPA1* KO U2OS cells and *OPA1* KO cells reconstituted with lenti-OPA1-YFP or lenti-OPA1^S158N^-YFP. Mean ± standard deviation from 3-4 independent experiments are shown (total of 55-83 cells). For panels D-G, n.s., no significance, ***P ≤ 0.001, **P ≤ 0.01, *P ≤ 0.05, assessed by one-way ANOVA. **(H–J)** Clinical data was mined from the St. Jude LIFE database to assess (H) body fat percentage, (I) blood triacylglycerol levels, and (J) blood cholesterol levels in patients with different S158N genotypes. **(K)** Proposed model of OPA1’s functions and impacts on LDs and mitochondria depending on its alternative targeting and its implication in human metabolic outcomes. OPA1-mediated lipase recruitment to LDs is indicated with a dashed arrow. Abbreviations: FAO, fatty acid oxidation; MTS, mitochondria targeting sequence.

The most common polymorphism in the OPA1 coding sequence, a substitution of serine to asparagine at position 158 (p.Ser158Asn; c.473G>A, rs7624750), is located within exon 4 and has an average allele frequency of 0.4710 (Table S1). The replacement with an asparagine at position 158 is accompanied by a relaxation of the N-terminal of the amphipathic helix as predicted by AlphaFold 3 (Fig. 7A), which further separates leucine^156^ from the rest of the non-polar residues in exon 4, possibly leading to weakened LD binding. Indeed, we observed a significant reduction in the relative enrichment of OPA1^S158N^-YFP on LDs (Fig. 7B, C), mimicking the defective LD interactions of OPA1^LFAA^ with synthetic mutations. Thus, we wondered if OPA1^S158N^ was also functionally distinct from OPA1 in FA release and mitochondrial characteristics. When repeating the OA withdrawal experiment, OPA1^S158N^ failed to rescue defects in FA release from LDs following Triacsin C treatment in *OPA1* KO U2OS cells (Fig. 7D). However, OPA1^S158N^ rescued mitochondrial morphology and connectivity regardless of the presence of LDs, contrasting the minimal rescue of OPA1 provided in LD-laden *OPA1* KO cells (Fig. 7E-G). As OPA1^S158N^ is a natural variant and OPA1^LFAA^ is a synthetic mutant, these observations further strengthened the idea that exon 4-mediated dynamic distribution of OPA1 on LDs plays a crucial role in releasing FAs from LDs and in regulating mitochondrial morphology and connectivity. Overall, these findings suggest OPA1 dynamically integrates the functions of both LDs and mitochondria via alternative organelle targeting.

It has already been established that functional coupling between LDs and mitochondria within cells can influence organismal metabolism^57, 58^. As serine^158^ and asparagine^158^ differentially regulate LDs and mitochondrial functions, we speculated this polymorphism may also affect human metabolism. Using the St. Jude Lifetime Cohort Study of individuals with a history of childhood cancer diagnosed and treated at St. Jude Children’s Research Hospital between 1962 and 2012 who survived ≥5 years from diagnosis, we identified lipid-related metabolic traits strongly correlated with this polymorphism. The body fat content, though the signal was weaker and appeared most evident under the recessive model, was higher in patients homozygous for asparagine^158^ (A/A) than in those with G/G or G/A genotypes (Fig. 7H), suggesting greater cellular LD accumulation in these individuals. Intriguingly, the strongest and most consistent signal was seen for blood TAGs, for which the A allele was associated with lower levels across all four tested models, including additive, dominant, recessive, and 2-degree-of-freedom (2DF) analyses (Fig. 7I and Table S2). Across the other lipid-related traits, the A allele tended to be associated with lower total cholesterol (Fig. 7J), and lower LDL cholesterol, while the direction for HDL cholesterol was positive, which is consistent with a potentially more favorable lipid profile overall (Table S2). The association of A allele with lower TAG and cholesterol was unexpected, as these metrics are all often linked to body fat content^59-62^. This dissociation between body fat and blood TAG and cholesterol suggests that the OPA1 may exert pleiotropic effects through context-dependent differences in lipid droplet–mitochondrial integration across tissues and cell types. Although these asparagine^158^-associated metabolic traits were not clinically significant compared with WT (consistent with benign classification of this polymorphism by the gnomAD database); these findings support the theory that these polymorphisms have modest but detectable effects, with both potentially beneficial and unfavorable associations depending on the phenotype. Altogether, these findings suggest that the differential coordination of FA release from LDs and mitochondrial dynamics by asparagine^158^ polymorphism is clinically relevant as a metabolic modifier in pediatric cancer survivors.

## Discussion

FAs are essential biomolecules playing critical roles in energy production, lipid homeostasis, and signal transduction. Nearly all cell types can store FAs and utilize these stockpiles when needed; our findings reveal novel mechanisms governing the utilization of these FA stockpiles (Fig. 7K). We first demonstrated that a subpopulation of OPA1 is localized to LDs through the exon 4 mediated targeting. When localized to LDs, OPA1 promotes the recruitment of lipases, facilitating the consequent FA release. This interaction dynamically controls OPA1’s entry into mitochondria, where OPA1 influences mitochondrial fusion and connectivity. We have established a mechanistic link between mitochondrial morphology and LD homeostasis. Finally, we have uncovered that OPA1’s alternative organelle distribution can be modulated by a common polymorphism in exon 4; this has clinical significance, as several metabolic traits in pediatric cancer survivors were correlated to this polymorphism. Overall, these findings broaden our collective understanding of OPA1’s role in lipid homeostasis and organelle crosstalk, providing new insights on the dynamic interplay between FA storage and mitochondrial morphology. Recent discoveries revealing non-canonical roles for OPA1 in stress responses, ferroptosis, and transcriptional activation within antitumor immunity^63, 64^ emphasize the pressing need for a deeper mechanistic understanding of this protein. This timely study provides important insights that help explain these emerging findings.

Lipolysis has been extensively studied in adipose tissues, the primary body fat storage depot in animals, and PKA plays an essential role in this process via the inducible recruitment of ATGL and HSL to LDs through a series of phosphorylation events. While emerging evidence indicates the existence of lipolysis in a variety of non-adipose cell types^4, 15^, this canonical view of lipolysis becomes insufficient because PLIN1, one of the key effectors downstream of PKA, is only expressed in selective tissues while lipases are widely expressed. As OPA1 is also broadly expressed in many human tissues and OPA1-mediated FA release occurs independently of canonical lipolytic signaling, we provide a potential explanation for how non-adipose cell types dynamically regulate and utilize FAs within LDs.

The mechanism behind OPA1’s ability to facilitate lipase recruitment is a question that warrants further investigation, as ATGL and HSL’s association with LDs is subject to complex regulations. In addition to lipolytic stimulation that dynamically recruits and activates ATGL on LDs via interactions with CGI-58 in adipocytes^65, 66^, ATGL also binds LDs directly via a C-terminal hydrophobic domain^67, 68 69^ and can be delivered to LDs via the Arf1/COPI machinery^70-72^. Similarly, HSL recruitment to LDs is regulated by both PKA-dependent phosphorylation and PKA-independent LD-binding motifs^73, 74^. Beyond these mechanisms, the physical properties of LDs, such as their size and levels of lipid packing defects, can broadly influence protein targeting^75, 76^. OPA1 may regulate ATGL and HSL recruitment through any one of these mechanisms. Notably, we observed that both endogenous and exogenous OPA1 frequently localize on clusters of small LDs, which have been characterized by higher surface-to-volume ratio and associated with enhanced lipolytic activity^77^. Given OPA1’s established role in membrane remodeling^78, 79^, it is plausible that OPA1 enhances lipase recruitment by altering the physical nature of LDs, thus facilitating FA release. Supporting this idea, membrane-morphing endosomal sorting complexes required for transport (ESCRT) components have been implied to play a role in facilitating inter-organelle FA trafficking^80, 81^. Therefore, it will be important to determine whether LD-associated OPA1 possesses membrane-remodeling capabilities that contribute to lipase recruitment and FA release.

The data presented here may begin to clarify previous conflicting observations^16-22^ regarding mitochondrial morphology and LD abundance under distinct metabolic conditions and perturbations from a technical point of view. OPA1’s role in mediating FA release may have been previously unrecognized due to the technical challenges related to the study of the tightly coupled functional interactions between LDs and mitochondria. By developing new FA flux assays with acute pharmacological perturbations designed to specifically assess trafficking routes across LDs and mitochondria, we found that OPA1 actively regulates FA release from LDs and mitochondrial FAO while minimally influencing FA import into mitochondria. This indicates FA trafficking routes between organelles are not directly coupled and emphasizes the importance of rigorously dissecting individual pathways to untangle the specific mechanisms underlying mitochondrial morphology and LD metabolism. Additional factors, such as heterogeneity of metabolically distinct mitochondria^82^ and mitochondria-mediated lipogenesis^83^, should be considered and examined thoroughly to achieve a comprehensive understanding of these processes.

To gain further insights into the regulation of OPA1 alternative organelle targeting, it is important to interrogate the mechanisms underlying the exon 4 splicing event. Our current analysis from the GTEx database revealed that heart and skeletal muscle have a higher inclusion of exon 4, whereas adipose tissue has a lower inclusion. Because heart and muscle primarily rely on FAO for ATP production^84, 85^, these data suggest a potential correlation between exon 4 inclusion and FA utilization. At the protein level, how OPA1’s exon 4 (or OPA1 itself) senses metabolic cues within the cytoplasm to determine its organelle distribution remain unknown. Both pyruvate and FAs drive the TCA cycle via the acetyl-CoA intermediate, raising the possibility that OPA1 may respond to the levels of these metabolites and dynamically adjust its organelle distribution based on fuel sources. Studying polymorphisms within exon 4 may also provide additional clues behind the metabolic regulation in OPA1’s subcellular localization. According to the gnomAD database, several polymorphisms of low allele frequency, such as p.Ser177Ile and p.Ile152Asn (Table S1), may also alter the non-polar interface of the amphipathic helix and potentially affect OPA1’s interactions with LDs.

In addition, given that OPA1’s localization to LDs is determined by the competition between exon 4 and its MTS, it is important to understand whether OPA1’s mitochondrial translocation itself could be regulated. While our computational prediction suggested that OPA1 has a strong MTS, one study identified phosphorylation events at serine 21 and 22 in OPA1’s N-terminal region^86^, which may also modulate MTS strength. Furthermore, the MitoFates prediction algorithm^53^ suggests OPA1’s first 39 amino acids could function as a weak MTS, with a probability score of 0.509 (where 1 represents maximum confidence). Interestingly, similarly low MTS probabilities have been associated with inefficient mitochondrial import of ATFS-1 in *Caenorhabditis elegans*^87^.Given the functional importance of OPA1 at both LDs and mitochondria, further studies illuminating the mechanisms by which OPA1 differentially targets to organelles should be prioritized. Our current data on the asparagine^158^ polymorphism (A allele) implicates the importance of OPA1’s alternative organelle targeting via a functional trade-off and the integration between LDs and mitochondria. This functional trade-off likely underlines the differential metabolic traits associated with serine^158^ and asparagine^158^, as each provides specific metabolic benefits. Integrating experimental interrogation of OPA1’s natural polymorphisms, along with continued MTS interrogation and clinical data mining, will likely uncover additional roles for OPA1 in human health and physiology through its complex moderation of LDs, mitochondria, and their interactions under various cell states.

Together, these findings highlight OPA1 as a previously unrecognized coordinator for LD–mitochondria communication via alternative organelle targeting, and provide an orthogonal view of the functional coupling between LD and mitochondria beyond physical contact sites. We propose that OPA1 acts as a molecular integrator aligning mitochondrial dynamics with lipid storage and utilization, enabling energy adaptation in response to cellular physiological demands.

## Material and Methods

### Cell lines, transfection, and transduction

U2OS cells (HTB-96), HeLa (CCL-2) cells, and *OPA1* KO MEFs (CRL-2995) were purchased from American Type Culture Collection (ATCC); Huh7 (JCRB0403) cells were purchased from the Japanese Cancer Research Bank. The WT MEFs were generated previously^88, 89^. All cells were maintained in their respective base media supplemented with 10% fetal bovine serum (FBS) and 1X penicillin/streptomycin solution per vendors’ recommendation. The base media used were McCoy’s 5A (Cat# 16600082, Thermo Fisher Scientific) for U2OS cells, Eagle’s Minimal Essential Medium (EMEM) (Cat# 30-2003, ATCC) for HeLa cells, and high-glucose Dulbecco's Modified Eagle Medium (DMEM) (Cat# 11995-065, Thermo Fisher Scientific) for Huh7 cells and MEFs. Cells were routinely tested via the MycoAlert^®^ Plus Mycoplasma Detection kit and found free of mycoplasma contamination (Cat# LT07-703, Lonza). Transfection and transduction were performed 16–24 h after cell seeding. DNA plasmid transfection was performed using TransIT-LT1 (Muris Bio LLC) for HeLa cells and Lipofectamine 3000 (Thermo Fisher Scientific) for U2OS and Huh7 cells with 50–500 ng DNA for 16–20 h according to manufacturer’s instructions. To knock down OPA1, HeLa or U2OS cells were transfected with 25 nM scramble (Cat# D-001810-10), OPA1 siRNA (Cat# L-005273-00-0020), or custom designed siRNA targeting OPA1’s exon 4–5 junction (sense: 5' CCUCAGGUUCUCCGGAAGAUU 3', antisense: 5' UCUUCCGGAGAACCU GAGGUU 3') (Dharmacon) using TransIT-TKO (Mirus Bio LLC) according to the manufacturer’s instructions for 48 h. Lentiviral particles were transduced into cells in the presence of 10 μg/mL protamine sulfate 40–48 h prior to the experiment. Multiplicity of infection (MOI) used were: 5 for TOM20-Halo, 20–100 for OPA1-YFP isoforms and mutants, 50 for OPA1-mhYFP, and 100 for iOPA1-YFP isoforms.

### Generation of OPA1 KO U2OS cells

OPA1−/− U2OS cells were generated using CRISPR technology in the Center for Advanced Genome Engineering at St. Jude Children’s Research Hospital [St. Jude]. First, 500,000 U2OS cells were transiently transfected with precomplexed ribonuclear proteins (RNPs) consisting of 150 pmol of chemically modified sgRNA (CAGE2484.OPA1.g3 spacer – 5’-CCUAUUUAAAG AUAGUUCUC −3’, IDT) and 50 pmol of 3X NLS SpCas9 protein (St. Jude Protein Production Core). Then, 300 ng of pMaxGFP (Lonza) plasmid was added to the transfection mix and transfected via nucleofection (Lonza, 4D-Nucleofector^™^ X-unit) according to the manufacturer’s protocol using solution P3 and program CM-104 in a 20-μL cuvette. Five days post nucleofection, cells were single-cell sorted by flow cytometry at the St. Jude Flow Cytometry and Cell Sorting Shared Resource Center to enrich for GFP+ (transfected) cells into 96-well tissue culture treated plates containing pre-warmed McCoy’s 5A media. Cells were clonally expanded and screened for the desired modification (out-of-frame indels) via targeted amplicon sequencing using gene specific primers with partial Illumina adapter overhangs (CAGE2484.OPA1.DS.F– 5’-CTACACGACGCTCTTCCGATCTtggatttgtgcaat gcagtagccct-3’ and CAGE2484.OPA1.DS.R-5’-CAGACGTGTGCTCTTCCGATCT-ccttacctcagggctaacggtacagc-3’, overhangs shown in uppercase) as previously described^90^. Next-generation sequencing analysis of clones was performed using CRIS.py^91^. Final clones were authenticated using the PowerPlex^®^ Fusion System (Promega) performed by the Hartwell Center for Biotechnology at St. Jude. Final clones were tested using the MycoAlertTMPlus Mycoplasma Detection Kit (Lonza) and found to be negative for mycoplasma. U2OS cells were further validated by western blotting and immunostaining.

### Reagents

BODIPY 493/503 (Cat# D3922), BODIPY 665/676 (Cat# B3932), sodium pyruvate (Cat# 11360070), D-glucose (Cat# A2494001), trypsin-EDTA (Cat# 25200056), PBS (Cat# 10010023), FBS (Cat# A5669801), Hoechst (Cat# 62249), MitoTracker Red CMXRos (Cat# M7512), and MitoTracker Deep Red FM (Cat# M22426) were purchased from Thermo Fisher Scientific. AUTODOT^™^ Visualization Dye, MDH (Cat# SM1000a) was purchased from Abcepta, oleic acid–BSA complex, hereafter oleic acid (OA), was from Sigma-Aldrich (Cat# O3008) and Cayman Chemicals (Cat# 29557). TopFluor-oleic Acid (Cat# 810259) and NBD-arachidonic acid (Cat# 810106) were purchased from Avanti Research. BODIPY-linoleic acid (Cat# 29795), DGAT1 inhibitor (PF-04620110, Cat# 16425), DGAT2 inhibitor (PF-06424439, Cat# 17680), and Triacsin C (Cat# 10007448) were from Cayman Chemical. FCCP (Cat# C2920-10MG), NG-497 (Cat# SML3712-5MG), lalistat-2 (Cat# SML2053-5MG), and 2-deoxy-D-glucose (Cat# D8375-10MG) were purchased from Sigma-Aldrich. Doxycycline was purchased from Takara (Cat# 631311). HaloTag ligand JF646 (Cat# HT1060) was purchased from Promega.

### DNA plasmids

YFP-tagged OPA1 (OPA1-YFP) was generated by inserting the Opa1 fragment amplified from U2OS cDNA into the pEYFP-N1 (Takara) plasmid between the SalI and BamHI sites. OPA1-YFP lentiviral plasmid (lenti-OPA1-YFP) was generating by inserting the OPA1-YFP fragment cut from the OPA1-YFP plasmid into the SJL12-CMV-GFP lentiviral backbone (provided by the St. Jude Vector Laboratory) between the XhoI and NotI restriction sites, followed by the removal of the Kozak sequence immediately 5’ of the YFP by site-directed mutagenesis. OPA1 isoform 2 and OPA1 mutants were generated by site-directed mutagenesis using lenti-OPA1-YFP as the template. To generate inducible OPA1-YFP lentiviral plasmids, a GFP-free TRE-mCh-E-GFP3G backbone (a Tet-on inducible plasmid provided by the St. Jude Vector Laboratory) was generated first by site-directed mutagenesis, followed by OPA1-YFP or OPA1^iso2^-YFP fragment insertion between the BlpI and BsiWI sites using InFusion to create TRE-iOPA1-YFP-E3G and TRE-iOPA1^iso2^-YFP-E3G, respectively. YFP-tagged OPA1 exon4 (exon 4-YFP) was generated by inserting a gblock containing the exon 4 coding sequence (synthesized by IDT, Newark, NJ) into pEYFP-N1 between the SalI and BamHI restriction sites, followed by the removal of Kozak sequence immediately 5’ of the YFP by site-directed mutagenesis. Exon 4-YFP mutants were generated by site-directed mutagenesis. Monomeric hyperfolder-YFP (mhYFP)-tagged OPA1 exon 4 was generated by replacing YFP in Exon 4-YFP with a mhYFP gBlock using BamHI and BsrGI restriction sites. OPA1-mhYFP was cloned by replacing YFP in the OPA1-YFP plasmid with a mhYFP gBlock using the AgeI and BsrGI restriction sites. Lenti-OPA1-mhYFP was subsequently generated by InFusion with an OPA1-mhYFP PCR fragment and a linearized SJL12-CMV-GFP backbone digested by XhoI and NotI restriction enzymes. Lenti-ATGL^S47A^-Halo was generated by inserting an ATGL PCR fragment amplified from U2OS cDNA and a Halo fragment into SJL12-CMV-GFP backbone using BsiWI, AgeI, and NotI restriction sites, followed by replacing S^47^ with A by site-directed mutagenesis. To generate mApple-CGI-58, CGI-58 fragment was amplified from U2OS cDNA and inserted into the mApple-C1 backbone plasmid using the HindIII and BamHI restriction sites. pEGFP-C1-PLIN 2 plasmid was purchased from Addgene (Plasmid #87161). Lenti-TOM20-Halo plasmid was generated by Genscript (Piscataway, NJ, USA) using pGenLenti backbone and a FLASH gene synthesis fragment containing a TOM20-Halo coding sequence from Halo-TOM20-N-10 (Addgene plasmid # 123284). Production and titration of lentivirus were performed by St. Jude Vector Laboratory as described previously^92, 93^. Oligonucleotides used for cloning can be found in Table S3. All plasmids used in this work were confirmed by sequencing.

### Generation of cDNA, PCR, and real-time PCR

Total RNA from U2OS and HeLa cells were extracted using the PureLink^™^ RNA Mini Kit (Thermo Fisher Scientific) according to the manufacturer’s instructions. Equal amounts of RNA (0.5–1 μg) were reverse-transcribed into cDNA using the SuperScript^™^ IV First-Strand Synthesis System Kit (Thermo Fisher Scientific) with oligo-dT primer. Quantitative real-time PCR was performed on the QuantStudio 6 Pro Real-Time PCR System using the PowerUp^™^ SYBR^™^ Green Master Mix Kit (Thermo Fisher Scientific). Oligonucleotides used for real-time PCR are listed in Table S3.

### Metabolomics and lipidomic analysis

U2OS WT and *OPA1* KO cells were grown in a 15-cm dish to full confluency, scraped and harvested in cold PBS. Cell pellets were snap-frozen in liquid nitrogen and stored in −80°C before shipping to Metabolon (Morrisville, NC, USA) for metabolomic and lipidomic analysis. For metabolomics analysis, samples were prepared using the automated MicroLab STAR^®^ system from Hamilton Company. Several recovery standards were added prior to the first step in the extraction process for quality control (QC) purposes. To remove protein, dissociate small molecules bound to protein or trapped in the precipitated protein matrix, and to recover chemically diverse metabolites, proteins were precipitated with methanol and vigorous shaking for 2 min (Glen Mills GenoGrinder 2000) followed by centrifugation at 500 g for 10 min at 12°C. The resulting extract was divided into equal fractions: two for analysis by two separate reverse phase (RP)/UPLC-MS/MS methods with positive ion mode electrospray ionization (ESI), one for analysis by RP/UPLC-MS/MS with negative ion mode ESI, one for analysis by HILIC/UPLC-MS/MS with negative ion mode ESI, while the remaining fractions were reserved for backup. Samples were placed briefly on a TurboVap^®^ (Zymark) to remove the organic solvent. All methods utilized a Waters ACQUITY ultra-performance liquid chromatography (UPLC) and a Thermo Scientific Q-Exactive high resolution/accurate mass spectrometer interfaced with a heated electrospray ionization (HESI-II) source and Orbitrap mass analyzer operated at 35,000 mass resolution^94^. The dried sample extract was then reconstituted in solvents compatible to each of the following four methods. Each reconstitution solvent contained a series of standards at fixed concentrations to ensure injection and chromatographic consistency.

One (1) aliquot was analyzed using acidic positive ion conditions, chromatographically optimized for more hydrophilic compounds (PosEarly). In this method, the extract was gradient eluted from a C18 column (Waters UPLC BEH C18-2.1x100 mm, 1.7 μm) using water and methanol, containing 0.05% perfluoropentanoic acid (PFPA) and 0.1% formic acid. The second (2) aliquot was also analyzed using acidic positive ion conditions; however, it was chromatographically optimized for more hydrophobic compounds (PosLate). The extract was gradient eluted from the same aforementioned C18 column using methanol, acetonitrile, water, 0.05% PFPA, and 0.01% formic acid; the column was operated at an overall higher organic content. The third (3) aliquot was analyzed using basic negative ion optimized conditions using a separate dedicated C18 column (Neg). The basic extracts were gradient eluted from the column using methanol and water with 6.5 mM ammonium bicarbonate at pH 8. The fourth and final (4) aliquot was analyzed via negative ionization following elution from a hydrophilic interaction chromatography (HILIC) column (Waters UPLC BEH Amide 2.1x150 mm, 1.7 μm) using a gradient consisting of water and acetonitrile with 10 mM ammonium formate, pH 10.8 (HILIC). The MS analysis alternated between MS and data-dependent MSn scans using dynamic exclusion. The scan range varied slightly between methods but covered 70–1000 m/z. Raw data was extracted, peaks identified and QC processed using a combination of Metabolon-developed software services (applications), and peaks were quantified using area-under-the-curve.

For lipidomics analysis, samples were homogenized in deionized water. About 11% of each homogenate was reserved for Bradford (protein) and/or DNA quantification for normalization purposes. The rest of each homogenate was subjected to a modified Bligh-Dyer extraction using methanol/water/dichloromethane in the presence of internal standards. The extracts were concentrated under nitrogen and reconstituted in 0.25 mL 10 mM ammonium acetate dichloromethane:methanol (50:50). The extracts were transferred to inserts and placed in vials for infusion-MS analysis, performed on a Shimazdu LC with nano PEEK tubing and the Sciex SelexIon-5500 QTRAP. The samples were analyzed via both positive and negative mode electrospray. The 5500 QTRAP scan was performed in multiple reaction monitoring (MRM) mode with the total of more than 1,100 MRMs. Individual lipid species were quantified by taking the peak area ratios of target compounds and their assigned internal standards, then multiplying by the concentration of internal standard added to the sample. Lipid species concentrations were background-subtracted using the concentrations detected in process blanks (water extracts) and run day normalized (when applicable). The resulting background-subtracted, runday normalized lipid species concentrations were then used to calculate the lipid class and FA total concentrations, as well as the mol% composition values for lipid species, lipid classes, and FAs.

### FAO analysis

FAO in U2OS cell was measured as described previously^95^. WT (50,000 cells/well) or *OPA1* KO (75,000 cells/well) cells were cultured in a volume of 0.5 mL/well in a 24-well plate. Three days after seeding, cells were visually confirmed to have a confluent monolayer. To measure FAO, a prewarmed mixture of 1 mM L-carnitine, 0.3% BSA, 100 μM oleate, and 0.4 μCi ^14^C-oleate in serum-free McCoy’s 5A media with or without 5 μM of Triacscin C was added to the cells. After a 3-h incubation at 37°C, 400 μL supernatant was added to 1.7-mL tubes for the collection of complete and incomplete FAO. Complete (^14^CO_2_ adsorbed on 1 M NaOH-soaked Whatman paper in cap) and incomplete FAO (soluble in 1 M perchloric acid) were measured with a Tri-Carb 2910TR liquid scintillation analyzer (Perkin-Elmer) for 3 min per vial in 3 mL Ultima Gold scintillation fluid (Sigma-Aldrich).

### Subcellular fractionation and western blotting

For subcellular fractionation, 6x10^6^ (per dish) U2OS cells were seeded in 15-cm dishes 24 h prior to 500 μM OA treatment for another 48 h. OA-treated cells were harvested and lysed in cold hypotonic lysis medium buffer (20 mM Tris-HCl, pH 7.4, 1 mM EDTA, pH 8.0) supplemented with proteinase inhibitor cocktail. Cellular fractions were isolated as previously described^93, 96^. Protein concentration of subcellular fractions and clear lysates were quantified using the Pierce BCA Protein Assay (Thermo Fisher Scientific). Equal amount of protein was denatured in 1x SDS-loading buffer at 95°C for 5–10 min and was loaded into an SDS-PAGE protein gel before being transferred to a nitrocellulose (Bio-Rad) or PVDF (Bio-Rad) membrane for western blotting.

For western blotting, membranes were blocked with EveryBlot blocking buffer (Bio-Rad) for 1 h before being incubated with a primary antibody at room temperature for 1 h or at 4°C for overnight. After incubation, the membranes were then washed with PBST for 10 min three times and incubated with a corresponding HRP conjugated secondary antibodies (1:3,000). The membranes were then washed again with PBST for 10 min three times before incubated with ECL substrates (Bio-Rad) and imaged with ChemiDoc (Bio-Rad) for chemiluminescence. Primary antibodies for western blot were used at a 1:2000 dilution, which included anti-OPA1 antibody (Cat# 80471S, Cell Signaling Technology), anti-ADFP (Plin2) antibody (Cat# AP5811c; Abcepta), anti-prohibitin B (PHB) antibody (Cat# PA5-19556, Invitrogen), anti-ATGL antibody (Cat# 2439, Cell Signaling Technology), anti-HSL antibody (Cat# 4107T, Cell Signaling Technology), anti-CGI-58 antibody (Cat# 183739, Abcam), anti-GAPDH antibody (Cat# 2118; Cell Signaling Technology), and anti-DGAT1 antibody (Cat# ab181180, Abcam). Secondary antibodies used were goat anti-rabbit IgG-HRP (Cat# 1706515, Bio-Rad) and goat anti-mouse IgG-HRP (Cat# 1706516, Bio-Rad).

### Immunostaining

Cells were fixed and immunostained as described previously^93^. All procedures were performed at room temperature while avoiding light, and all wash steps were using PBS unless otherwise indicated. First, cells were rinsed and fixed with 4% paraformaldehyde (PFA, Electron Microscopy Sciences) and 0.05% glutaraldehyde (GA, Electron Microscopy Sciences) in PBS for 15 min. Fixed cells were washed three times and permeabilized with 0.3% Triton X-100 in PBS for 20 min. Permeabilized cells were then blocked with 5% normal donkey or goat serum in PBS for 1 h followed by incubation with primary antibody (1:200 in dilution; anti-OPA1 antibody, Cat# 67589, Cell Signaling Technology; anti-PLIN 2 (Cat# AP5118c, Abcepta); anti-TOM20 antibody (Cat# ab186735 or ab56783, Abcam) in PBS with 1% BSA (Sigma-Aldrich) at 4°C overnight. The next day, the samples were washed three times before being incubated with a secondary antibody, including Alexa Fluor Plus 488 conjugated donkey anti-rabbit IgG (Cat# A32790), Alexa Fluor Plus 555 conjugated goat anti-rabbit IgG (Cat# A32732), or Alexa Fluor Plus 647 conjugated goat anti-rabbit IgG (Cat# A32733, Thermo Fisher Scientific) for 1 h. The stained samples were washed three times and imaged using confocal microscopy.

### Fluorescence microscopy imaging, electron microscopy, and image analysis

All imaging experiments were performed on a custom-built Nikon microscope equipped with a Yokogawa CSU-W1 Spinning Disk unit, Z-axis piezo nanopositioners, and Tokai Hit STXG CO_2_ incubation system using a 60x (CFI APO TIRF, NA 1.49 oil) objective. Unless otherwise indicated, all cells were grown and transiently transfected with plasmids or transduced with lentiviral particles on #1.5 cover glasses imaging vessels, including Lab-Tek II chambered cover glasses (Cat# Z734853-96EA, Thermo Fisher Scientific) and MatTek dishes (Cat# P35G-1.5-10-C, MatTek). Prior to live-cell imaging, media were replaced with Fluoro-Brite DMEM (Thermo Fisher Scientific) supplemented with 5% FBS and penicillin/streptomycin and then imaged at 37°C supplemented with 7% CO_2_ in humidified air. Immunostaining samples were imaged in PBS at room temperature. All image analysis were performed with Fiji^97^ unless otherwise indicated. For intensity-based analysis, all intensity-based analyses were subjected to background (area without cells) subtraction.

#### OA withdrawal.

To evaluate the FA release from LDs, cells seeded in 8-well chambered cover glass with transfection, transduction, and/or perturbation were treated with 100 μM OA overnight to induce LD formation. The next day, OA-containing media were replaced with fresh OA-free media with 10 μM Triacsin C or without glucose to induce FA release from LDs. Cells were then stained with BODIPY-493/503 (500 ng/mL) or MDH (20 μM) to visualize LDs in imaging media prior to imaging. Confocal z-stacks were taken to sample total number of LDs throughout cells. To quantify total LD content, individual cells were randomly selected, and maximal intensity projections (MIPs) were created from z-stacks. Total LD area was segmented by intensity thresholding of BODIPY or MDH and measured using ‘Analyze Particles’ in Fiji. Relative LD contents were obtained by normalizing all data to the average of controls.

#### Mitochondrial FA import.

WT and *OPA1* KO U2OS cells grown on 8-well chambered cover glass transduced with TOM20-Halo were treated with 20 μM of PF-04620110 (DGAT1 inhibitor) and 20 μM PF-06424439 (DGAT2 inhibitor) simultaneously or with 10 μM Triacsin C in imaging media in the presence of 100 nM JF646 for 30 min to blunt LD biogenesis or mitochondrial FA trafficking, respectively. Without changing media, treated cells were then incubated with 1 μM TopFluor-OA, 1 μM BODIPY-linoleic acid, or 1 μM NBD-arachidonic acid for 10 min prior to imaging. Z-stack images from the bottom half of the cells were taken. To quantify mitochondrial FA import, MIPs from three continuous images from the bottom were first generated in Fiji. Areas at the cell periphery were cropped (examples in Figure 1E) to avoid out-of-focus signals in the peri-nuclear regions. Mitochondria masks were created by thresholding the TOM20-Halo channel and mean FA intensity within masks were measured.

#### Relative enrichment of OPA1 on LD.

To image OPA1 isoforms and mutant, cells grown in complete basal media on imaging cover glasses for 24 h were transduced with OPA1-expressing lentivirus. One day later, transduced cells were incubated with fresh media containing indicated FAs for another 20 h. Prior to imaging, cells were stained with 200 nM MitoTracker DeepRed for 3–5 min followed by media change to imaging media containing an LD stain (BODIPY or MDH). For the inhibitor experiment, 20 μM FCCP or 4 mM 2-DG was added during media change a day after transduction. For the experiment using media with modified pyruvate and/or glucose levels, cells were grown and transduced with OPA1-expressing lentivirus in their complete basal media, followed by media replacement with 100 μM OA and indicated concentrations of pyruvate and glucose for 20 h prior to imaging. To quantifying relative protein enrichment on LDs, confocal z-stack images of OPA1 isoforms and mutants as well as exon 4 and mutants were acquired with the Nikon confocal microscope together with LD staining and/or MitoTracker. MIPs of the LD channel of were used to create LD masks via thresholding, which was then applied to the OPA1 channel using the ROI manager for LD mean intensity (mean_LD_) measurement. Mean intensity of the whole cell (mean_cell_) or of regions devoid of LDs (mean_cyt_) were also quantified for expression level normalization.

#### Relative enrichment of ATGL and CGI-58 on LDs.

For ATGL imaging, U2OS cells were seeded onto imaging cover glass one day prior to transduction with ATGL S47A-Halo lentivirus at an MOI of 10–40. Cells were treated with OA 24 h after transduction and imaged with the Nikon confocal microscope 40 h later. For CGI-58 imaging, U2OS cells were transfected with 50 ng mApple-CGI-58 plasmid, treated overnight with 100 μM OA, and imaged the following day. Quantification of the relative enrichment of ATGL and CGI-58 on LDs were performed similarly to the analysis of OPA1 described above.

#### Inducible production of OPA1.

*OPA1* KO U2OS cells were plated in 8-well chambered coverglass and transduced with TRE-OPA1-YFP-E3G or TRE-OPA1^iso2^-YFP-E3G lentivirus at an MOI of 100. After 48 h, transduced cells were incubated with 100 μM OA for 8 h to induce LD formation, followed by staining with 200 nM MitoTracker Red CMXRos for 5–10 min. Time-lapse imaging was conducted in 30-min interval for 14 h in imaging media containing BODIPY-665/676 to stain LDs and 4 μg/mL doxycycline to induce OPA1 production. For the experiment looking at the absence and presence (−/+) of pyruvate, OPA1 U2OS KO cells were transduced with TRE-OPA1-YFP-E3G for 24 h, followed by a change of media containing with 100 μM OA and 0–1 mM pyruvate. Approximately 20 h later, 4 μg/mL doxycycline was added for 4–5 h with indicated levels of pyruvate to induce OPA1-YFP expression prior to imaging. OPA1-YFP or OPA1^iso2^-YFP signal within individual cells were categorized into: (i) mitochondria, (ii) LDs, or (iii) mitochondria and LDs, based on their subcellular distribution.

#### Mitochondrial morphology analysis.

U2OS WT, *OPA1* KO, and *OPA1* KO cells reconstituted with OPA1 mutants across indicated conditions were stained with 200 nM MitoTracker Deep Red for 3 min. Cells were washed in warm McCoy’s 5A medium three times before Z-stack images were acquired. To quantify mitochondrial morphological features, MIPs were generated from three continuous bottom images and analyzed using Fiji’s ‘Mitochondria-Analyzer’ plug-in^54^. The 2D analysis mode was used with a block size of 1.350 microns and C-value of 5.

#### Correlative confocal-scanning electron microscopy.

Four thousand U2OS cells in 300 μL McCoy’s 5A medium were plated onto the grided cover glass area within MatTek dishes (Cat# P35G-1.5-14-C-GRID, MatTek). Twenty-four hours after plating, cells were transduced with lenti-OPA1-mhYFP at an MOI of 50. Twenty-four hours after transduction, the media was changed to include 100 μM OA and left to incubate overnight. Alternatively, for the exon 4 experiment, cells were transfected with 150 ng exon4-hfYFP plasmid 48 h after plating in fresh media with 100 μM OA overnight. Transduced or transfected cells were sequentially stained with 400 nM MitoTracker Red CMXRos and 20 μM Hoechst at 37°C for 5 min, followed by staining with 500 ng/mL BODIPY 665 at 37°C for 30 min. After staining, cells were fixed with 2.5% glutaraldehyde and 2% PFA fixation in 0.1 M sucrose prepared in 0.1 M Sorenson’s phosphate buffer (PB) at room temperature for 30 min. Fixed cells were rinsed before imaging in PBS to acquire z-stack fluorescence images and locate cells of interest.

After confocal imaging, cells were further fixed in 2.5% glutaraldehyde, 2% PFA, and 0.1 M sucrose in PB for 3 days at 4°C. The fixed cells were post-fixed in a mixture of 0.5% OsO_4_ + 0.5% K_4_Fe(CN)_6_ in 0.1 M PB for 10 min on Lab Armor beads pre-chilled to 4°C in a Styrofoam cooler for lipid fixation and contrasting. The post-fixed cells were further contrasted with 1% uranyl acetate in H_2_O overnight at 4°C. Cells were dehydrated with an ascending ethanol series (10%, 30%, 50%, 70%, 80%, 90%, 95%, and 100%), with each step carried out for 5 min on cold beads, before the cells were embedded in Spurr’s resin (Cat#14300, Electron Microscopy Sciences). The resin-embedded cells were thermally polymerized for 24 h at 70°C. After thermal polymerization, all cells, including the target cells, were transferred to the polymerized resin side of the MatTek dish without any physical damage once the glass cover slip was removed.

Following transfer, the target cells were serially sectioned to a dimension of 300 μm x 300 μm x 100 nm on a Leica ARTOS 3D ultramicrotome using a DiATOME Histo Jumbo diamond knife. Approximately 40 serial sections with the desired dimension were collected on a silicon chip (5 mm x 7 mm) and dried for 1 h at 45°C. The serial sections were coated with 6 nm carbon in a Leica EM ACE600 High Vacuum Coater before scanning electron microscope (SEM)-based array tomography imaging. The target cells were imaged in a Zeiss GeminiSEM 460 using a Sense backscattered electron detector with 1.5 kV high tension and 80 pA current. Zeiss Atlas 5 Array Tomography software assisted in the automatic selection of the target cells throughout the serial sections and performed high-resolution imaging at 2-nm pixel resolution in a 30 μm x 30 μm of field of view. The obtained images from serial sections were aligned using the lipid droplets as internal fiducials and exported as tagged image file format (TIFF) files by Atlas 5 Array Tomography to co-register to the obtained confocal microscopic images.

### Prediction of OPA1 MTS strength

The MTS of OPA1 was tested for its relative strength by calculating its amphiphilicity as described^53^. Amphiphilicity was calculated by defining the net charge of the OPA1 MTS at pH 7.4 (calculated at ProtPI, https://www.protpi.ch/Calculator/ProteinTool) and its maximal hydrophobic moment (calculated at EMBOSS, https://www.bioinformatics.nl/cgi-bin/emboss/hmoment) and multiplying these two values. This amphiphilicity value was compared with experimentally determined parameters defining MTS strength for 12 independent constructs, as reported^53^.The OPA1 MTS was defined based on a previous study^28^ and was additionally defined through the MitoFates database (https://mitf.cbrc.pj.aist.go.jp/MitoFates/cgi-bin/top.cgi).

### GTEx data mining

All GTEx (V8) RNA-seq data were remapped to human genome reference GRCh38 using Gencode reference annotation with STAR (v2.7.9a)^98^ in a splice-aware manner. The junction read counts for *OPA1* Exon3-Exon4, Exon 4-Exon5 and Exon3-Exon5 were used to compute the percent of inclusion (PSI) of exon 4 and percent of exclusion (PSE).

### SJLIFE data analysis

The St. Jude Lifetime cohort participants return for a clinical visit that includes sample collection, germline genotyping and laboratory testing^99^.

This analysis focused on replication of association signals for a single germline variant, chr3:193617202:G>A (hg38), in five prespecified lipid related traits in SJLIFE: body fat percentage, blood triglycerides (TAG), total cholesterol, LDL cholesterol, and HDL cholesterol. Whole-exome sequencing data from 4,328 SJLIFE participants with genotype data were used as the starting dataset before trait-specific phenotype missingness was applied. The cohort was 52.1% male, and the mean age at sample collection was 27.99 years (SD 11.39; median 27.13).

For each of the five traits, we first evaluated whether analysis should be performed on the raw phenotype scale or after inverse-rank normal transformation (IRNT), based on the distribution of covariate-adjusted residuals. Raw values were retained only when absolute residual skewness <= 0.5 and absolute excess kurtosis value <= 1; otherwise IRNT was used. Under this rule, body fat percentage was analyzed on the raw scale, while TAG, total cholesterol, LDL cholesterol, and HDL cholesterol were analyzed on the IRNT scale.

We then tested four prespecified genetic models for each trait using PLINK2: additive (--glm), dominant (--glm dominant), recessive (--glm recessive), and genotypic 2-degree-of-freedom (2DF, --glm genotypic). Two analysis settings were considered: a crude analysis without covariates, and an adjusted analysis including AgeAtLastContact, AgeAtDiagnosis, Gender, ReceivedChemotherapy, and ReceivedRadiation. We did not include genetic principal components in these models, because this follow-up was restricted to replication of a single common variant with balanced allele frequency and not a genome-wide association analysis.

### Statistical analysis

Raw data points (simi-transparent symbols) and mean −/+ standard deviation from independent experiments (bold symbols and lines) were plotted together to simultaneously convey cell variability and experimental reproducibility. Mean values from independent experiments were statistically analyzed in GraphPad Prism (Version 11.0.0, GraphPad Software, Inc., San Diego, CA, USA) using unpaired *t*-test or one-way ANOVA. Graphs and figures were generated using GraphPad Prism and Adobe Illustrator (Version 29.8.5, San Jose, CA, USA).

## Supplementary Material

Supplement 1
