# Supplementary material for: Alternative organelle targeting of OPA1 mediates fatty acid release from lipid droplets
Source: bioRxiv. 2026 May 11:2026.05.07.723579. Preprint. [Version 1] doi: 10.64898/2026.05.07.723579 (PMC13192942; doi:10.64898/2026.05.07.723579)
Supplement: Supplement 1 [file NIHPP2026.05.07.723579v1-supplement-1.pdf]

bioRxiv preprint doi: https://doi.org/10.64898/2026.05.07.723579; this version posted May 11, 2026. The copyright holder for this preprint (which was not certified by peer review) is the author/funder, who has granted bioRxiv a license to display the preprint in perpetuity. It is made available under a CC-BY 4.0 International license.

Figure S1

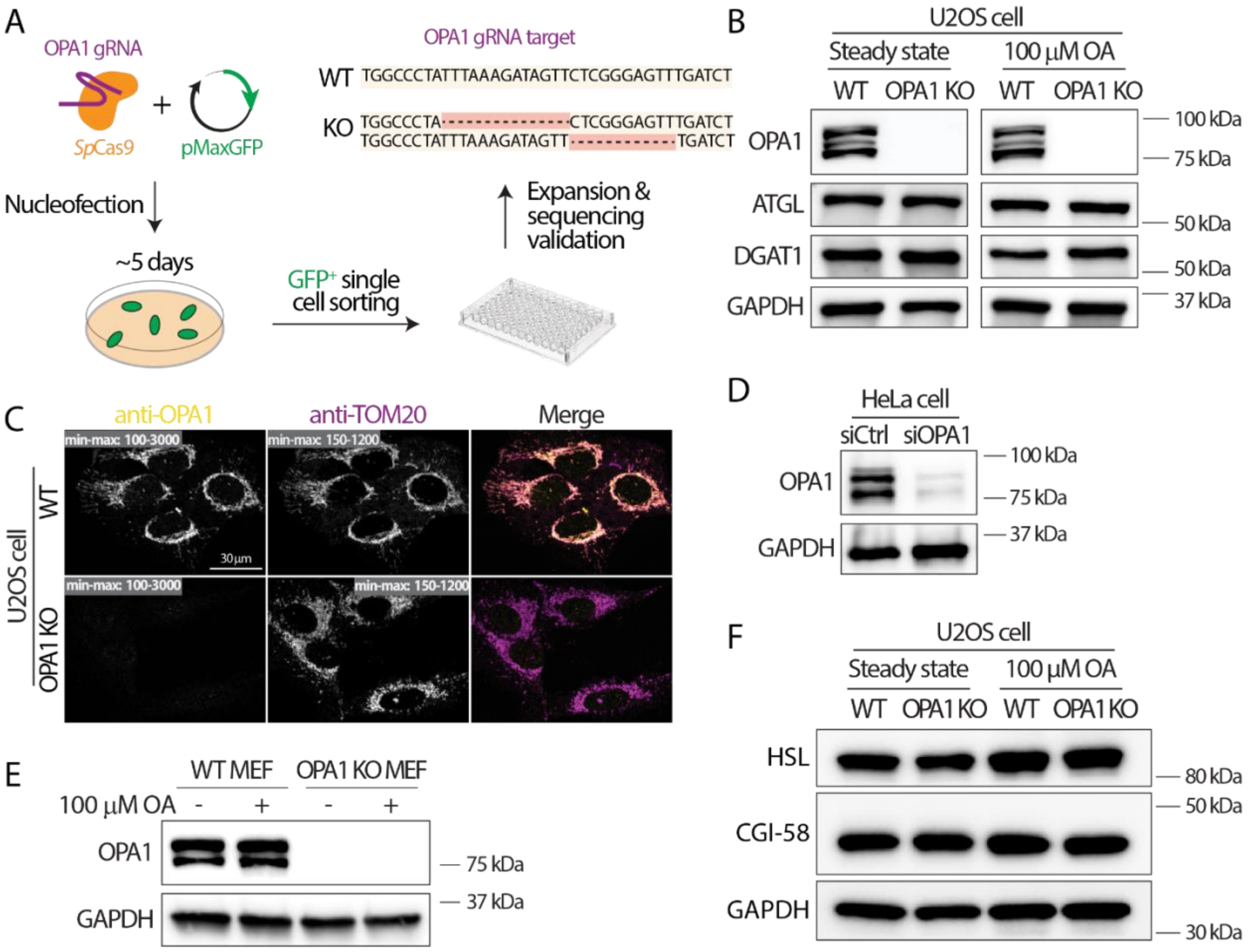

bioRxiv preprint doi: https://doi.org/10.64898/2026.05.07.723579; this version posted May 11, 2026. The copyright holder for this preprint (which was not certified by peer review) is the author/funder, who has granted bioRxiv a license to display the preprint in perpetuity. It is made available under a CC-BY 4.0 International license.

Figure S1. Generation and validation of OPA1 knockout and knockdown cells. (A) Diagram illustrating the generation of *OPA1* knockout (KO) U2OS cells via CRISPR-genome editing. Sequencing data validating *OPA1* KO is shown in the top right, and deleted *OPA1* genome sequences are represented as dashed lines highlighted in red. **(B)** Western blot analysis of OPA1, ATGL, DGAT1, and GAPDH in wildtype (WT) and *OPA1* KO U2OS cells -/+ overnight 100 μM oleic acid (OA) treatment. **(C)** Immunostaining of endogenous OPA1 and TOM20 in WT and *OPA1* KO U2OS cells detected by confocal microscopy. Maximal intensity projected confocal images from whole cells with min-max intensity range (gray boxes) are shown. **(D)** Western blot analysis of OPA1 and GAPDH in HeLa cells transfected with scramble siRNA (siCtrl) or OPA1 siRNA. **(E)** Western blot analysis of OPA1 and GAPDH in WT and *OPA1* KO mouse embryonic fibroblasts (MEFs) -/+ overnight 100 μM OA treatment. **(F)** Western blot analysis of HSL, CGI-58, and GAPDH in WT and *OPA1* KO U2OS cells -/+ overnight 100 μM OA treatment.

bioRxiv preprint doi: https://doi.org/10.64898/2026.05.07.723579; this version posted May 11, 2026. The copyright holder for this preprint (which was not certified by peer review) is the author/funder, who has granted bioRxiv a license to display the preprint in perpetuity. It is made available under a CC-BY 4.0 International license.

Figure S2

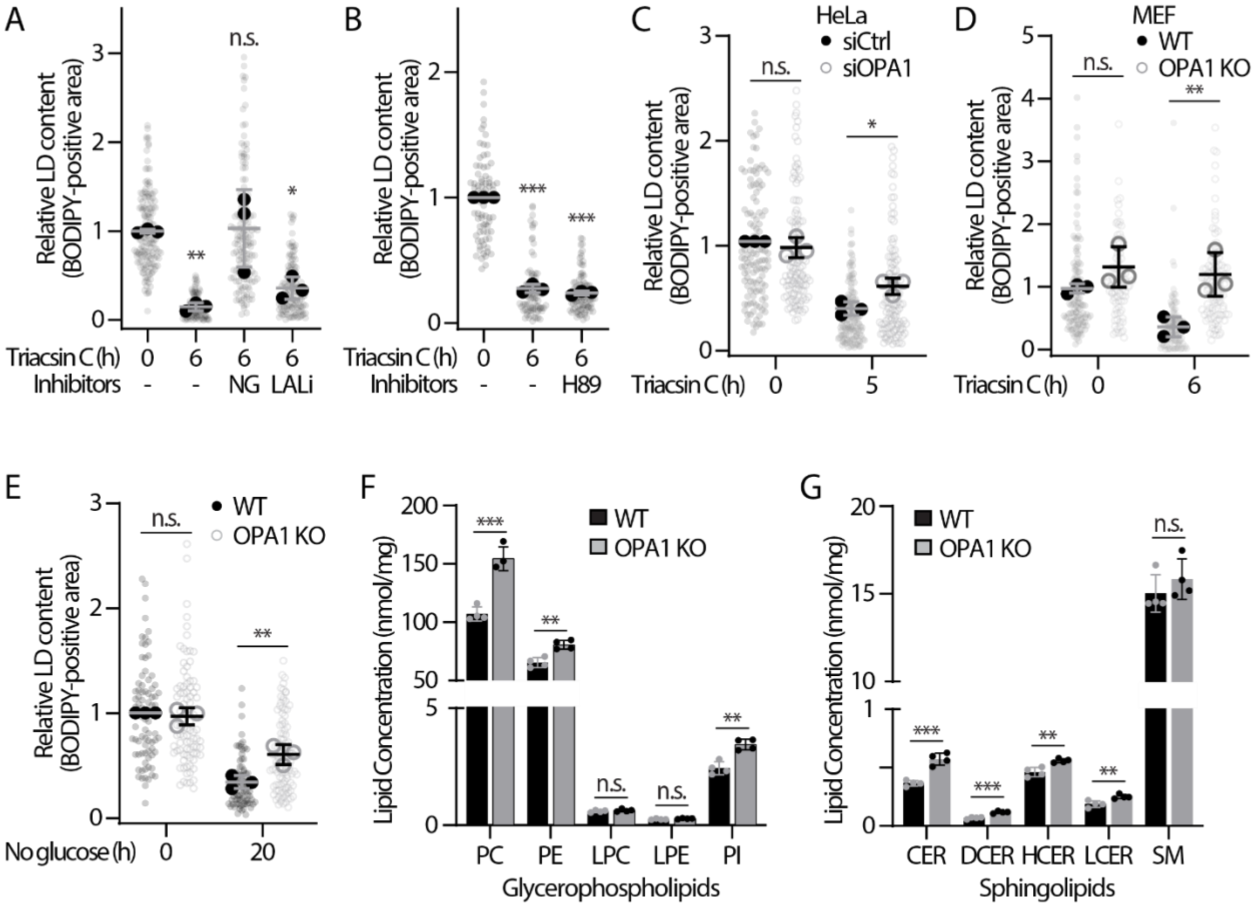

bioRxiv preprint doi: https://doi.org/10.64898/2026.05.07.723579; this version posted May 11, 2026. The copyright holder for this preprint (which was not certified by peer review) is the author/funder, who has granted bioRxiv a license to display the preprint in perpetuity. It is made available under a CC-BY 4.0 International license.

Figure S2. Characterization of the breakdown and biogenesis of lipid droplets and phospholipids in wildtype and *OPA1*-silenced cells. (A) BODIPY 493/503-positive area in U2OS cells treated with 100 μM oleic acid (OA) overnight followed by 10 μM Triacsin C incubation in the presence of 10 μM NG497 (NG) or 5 μM Lalistat 2 (LALi) for 6 h. Mean ± standard deviation from three independent experiments are shown (total of 129–148 cells). For statistics in panels A–E, n.s., no significance, ***$P \leq 0.001$, **$P \leq 0.01$, *$P \leq 0.05$, as assessed by one-way ANOVA. **(B)** BODIPY 493/503-positive area in U2OS cells treated with 100 μM OA overnight followed by 6-h co-incubation of 10 μM Triacsin C and 10 μM H89. **(C)** BODIPY 493/503-positive area in HeLa cells transfected with scramble siRNA (siCtrl) or *OPA1* siRNA (siOPA1) treated with 100 μM OA overnight before 6-h incubation with 10 μM Triacsin C. Mean ± standard deviation from three independent experiments are shown (total of 107–122 cells). **(D)** BODIPY 493/503-positive area in WT and *OPA1* knockout (KO) mouse embryonic fibroblasts (MEFs) treated with 100 μM OA overnight before 6-h incubation with 10 μM Triacsin C. Mean ± standard deviation from three independent experiments are shown (total of 83–105 cells). **(E)** BODIPY 493/503-positive area in WT and *OPA1* KO U2OS cells treated with 100 μM OA overnight followed by 20-h incubation in glucose-free Dulbecco's Modified Eagle Medium. Mean ± standard deviation from three independent experiments are shown (total of 79–87 cells). **(F and G)** Levels of (F) glycerophospholipids and (G) sphingolipids in steady-state WT and *OPA1* KO U2OS cells determined using liquid chromatography–mass spectrometry. Mean ± standard deviation from four replicates are shown. For statistics in panels F and G, n.s., no significance, ***$P \leq 0.001$, **$P \leq 0.01$, as assessed by unpaired *t*-test. Abbreviations: PC, phosphatidylcholine; PE, phosphatidylethanolamine; LPC, lysophosphatidylcholine; LPE, lysophosphatidylethanolamine; PI, phosphatidylinositol; CER, ceramide; DCER, dihydroceramide; HCER, hexosylceramide; LCER, lactosylceramide; SM, sphingomyelin.

bioRxiv preprint doi: https://doi.org/10.64898/2026.05.07.723579; this version posted May 11, 2026. The copyright holder for this preprint (which was not certified by peer review) is the author/funder, who has granted bioRxiv a license to display the preprint in perpetuity. It is made available under a CC-BY 4.0 International license.

Figure S3

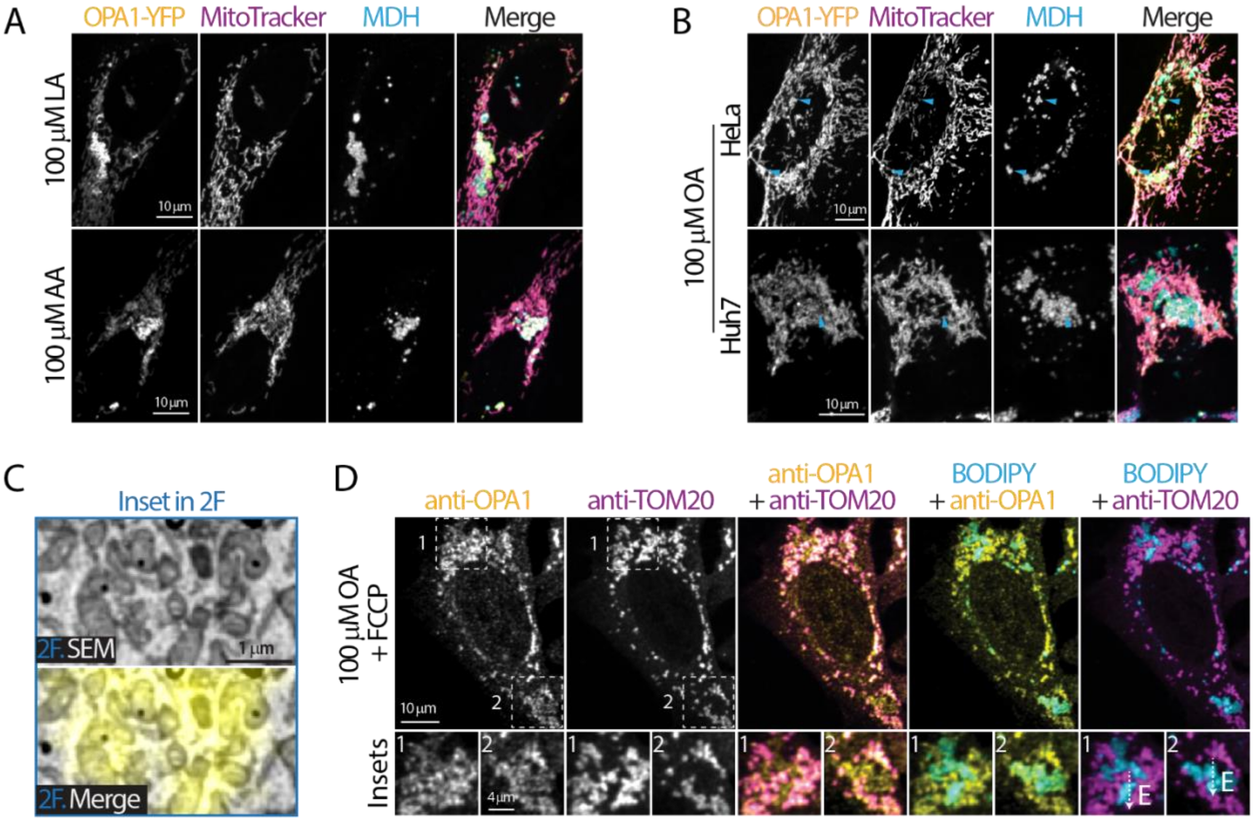

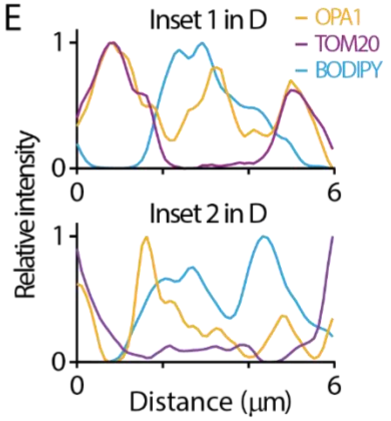

F

MTS	Species	Length (a.a.)	Charge	Max μH	Amphiphilicity	Import velocity (*k*)
Hsp60	*S. cerevisiae*	21	5.27	0.690	3.64	0.493
Atp2	*S. cerevisiae*	32	5.24	0.637	3.34	0.418
Mrp21	*S. cerevisiae*	24	4.16	0.695	2.89	0.317
Pim1	*S. cerevisiae*	37	8.26	0.669	5.53	0.394
Cit1	*S. cerevisiae*	37	4.20	0.630	2.65	0.321
Mdh1	*S. cerevisiae*	17	2.27	0.821	1.86	0.256
Mrpl36	*S. cerevisiae*	10	2.27	0.166	0.376	0.187
Atp2	*N. crassa*	44	7.27	0.727	5.29	0.493
ATFS-1	*C. elegans*	54	2.44	0.655	1.60	0.286
HSP-60	*C. elegans*	16	3.27	0.824	2.69	0.322
Mrpl36-n20	*S. cerevisiae*	20	2.26	0.549	1.24	0.233
Mrpl36-n31	*S. cerevisiae*	31	7.26	0.599	4.35	0.409
**OPA1**	*H. sapiens*	87	14.23	0.555	7.90	**0.611*

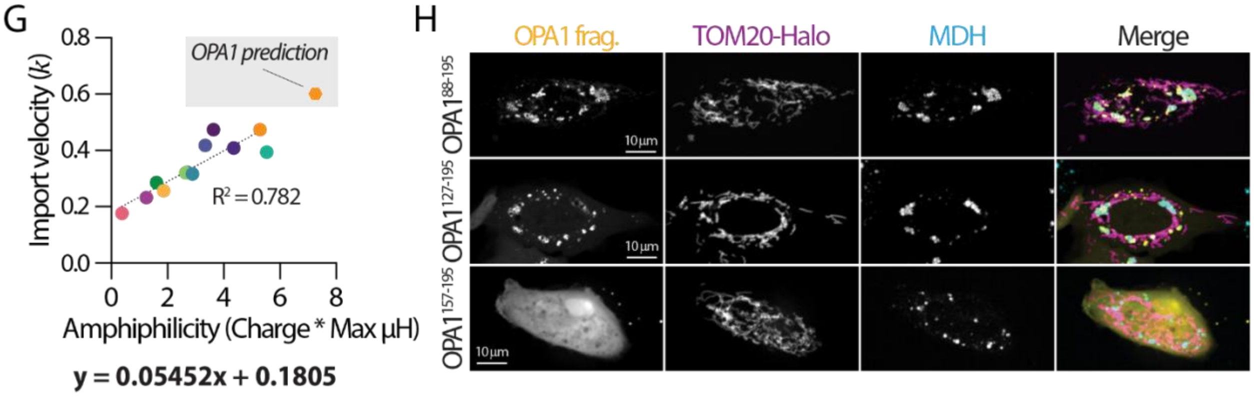

bioRxiv preprint doi: https://doi.org/10.64898/2026.05.07.723579; this version posted May 11, 2026. The copyright holder for this preprint (which was not certified by peer review) is the author/funder, who has granted bioRxiv a license to display the preprint in perpetuity. It is made available under a CC-BY 4.0 International license.

Figure S3. Localization of OPA1 and OPA1 fragments. (A) Localization of OPA1-YFP (lentiviral), mitochondria (labeled with MitoTracker Deep Red), and lipid droplets (LDs, labeled with MDH) in U2OS cells treated with 100 μM linoleic acid (LA) or 100 μM arachidonic acid (AA) overnight. Maximal intensity projected (MIP) confocal images from four axial slices (~1 μm total thickness) are shown. **(B)** Localization of OPA1-YFP, mitochondria (MitoTracker Deep Red), and LD (MDH) in HeLa and Huh7 cells treated with 100 μM oleic acid (OA) overnight, detected with confocal microscopy. MIP confocal images from four axial slices (~1 μm total thickness) are shown. Cyan arrowheads indicate area with LDs. (**C**) Inset from the correlative confocal-scanning electron microscopy (SEM) image in Fig. 2F, outlined in blue, showing OPA1-mhYFP localization in mitochondria in a U2OS cell treated with 100 μM OA overnight. **(D)** Subcellular localization of endogenous OPA1 (anti-Opa1) on mitochondria (anti-TOM20) and LDs (BODIPY 493/503) in a U2OS cell treated with 100 μM OA and 20 μM FCCP (an uncoupler of mitochondria oxidative phosphorylation) overnight and monitored via confocal microscopy. Sum of confocal images from five axial slices (~1.2 μm total thickness) are shown. **(E)** Relative intensity profiles of OPA1, TOM20, and BODIPY measured from lower right panel in (D), indicated by white-dashed arrows. **(F)** Properties of representative mitochondrial targeting sequences (MTSs) for mitochondrial protein import. Asterisk indicates the predicted import velocity of the OPA1 MTS. Max μH, maximal helical hydrophobic moment. **(G)** Correlation between the amphiphilicity of MTS and the protein import velocity from (F). **(H)** Localization of truncated YFP-tagged OPA1 fragments (frag.), mitochondria (labeled with TOM20-Halo; JF646), and LDs (MDH) in U2OS cells treated with 100 μM OA overnight and detected by confocal microscopy. MIP confocal images from four axial slices (~1 μm total thickness) are shown.

bioRxiv preprint doi: https://doi.org/10.64898/2026.05.07.723579; this version posted May 11, 2026. The copyright holder for this preprint (which was not certified by peer review) is the author/funder, who has granted bioRxiv a license to display the preprint in perpetuity. It is made available under a CC-BY 4.0 International license.

1 **Figure S4**

2

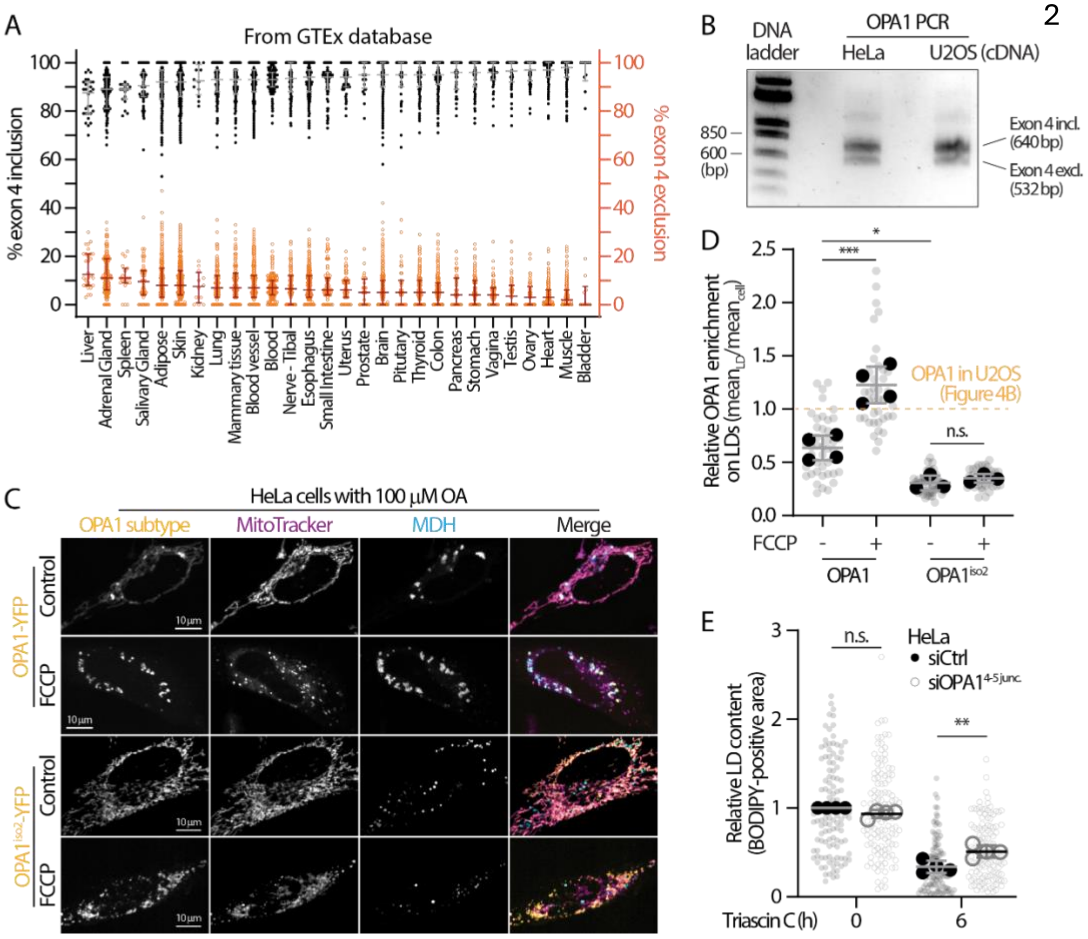

bioRxiv preprint doi: https://doi.org/10.64898/2026.05.07.723579; this version posted May 11, 2026. The copyright holder for this preprint (which was not certified by peer review) is the author/funder, who has granted bioRxiv a license to display the preprint in perpetuity. It is made available under a CC-BY 4.0 International license.

Figure S4. OPA1^{iso2} mRNA expression, protein localization, and effect on fatty acid release. (A) Percentage of OPA1 exon 4 inclusion and exclusion, representing OPA1 isoform 1 and isoform 2, respectively, across various human tissues analyzed from the Genotype-Tissue Expression (GTEx) database. Raw data and median values with quartile ranges are shown. **(B)** Profiling of OPA1 exon 4 inclusion (incl.) and exclusion (excl.) from cDNA of HeLa and U2OS cells. **(C)** Localization of OPA1-YFP or OPA1^{iso2}-YFP, mitochondria (labeled with MitoTracker Deep Red), and lipid droplets (LDs; labeled with MDH) in HeLa cells treated with 100 μM oleic acid (OA) -/+ 20 μM FCCP (an uncoupler of mitochondria oxidative phosphorylation) overnight and detected by confocal microscopy. Maximal intensity projected confocal images from four axial slices (~1 μm in total thickness) are shown. **(D)** Relative enrichment of OPA1 and OPA1^{iso2} on LDs as described in (C). Mean ± standard deviation from three-four independent experiments are shown (total of 31–42 cells). Yellow dashed line indicates the relative enrichment of OPA1 on LDs in U2OS cells as described in Figure 4B. n.s., no significance, ***P ≤ 0.001, *P ≤ 0.05, as assessed by one-way ANOVA. **(E)** BODIPY-positive LD content in siCtrl and siOPA1$^{4\text{-}5\ junc}$ transfected HeLa cells treated with 100 μM OA overnight followed by incubation with 10 μM Triacsin C for 6 h. Mean ± standard deviation from four independent experiments are shown (total of 107–122 cells). n.s., no significance, **P ≤ 0.01, as assessed by one-way ANOVA.

bioRxiv preprint doi: https://doi.org/10.64898/2026.05.07.723579; this version posted May 11, 2026. The copyright holder for this preprint (which was not certified by peer review) is the author/funder, who has granted bioRxiv a license to display the preprint in perpetuity. It is made available under a CC-BY 4.0 International license.

1 **Figure S5**

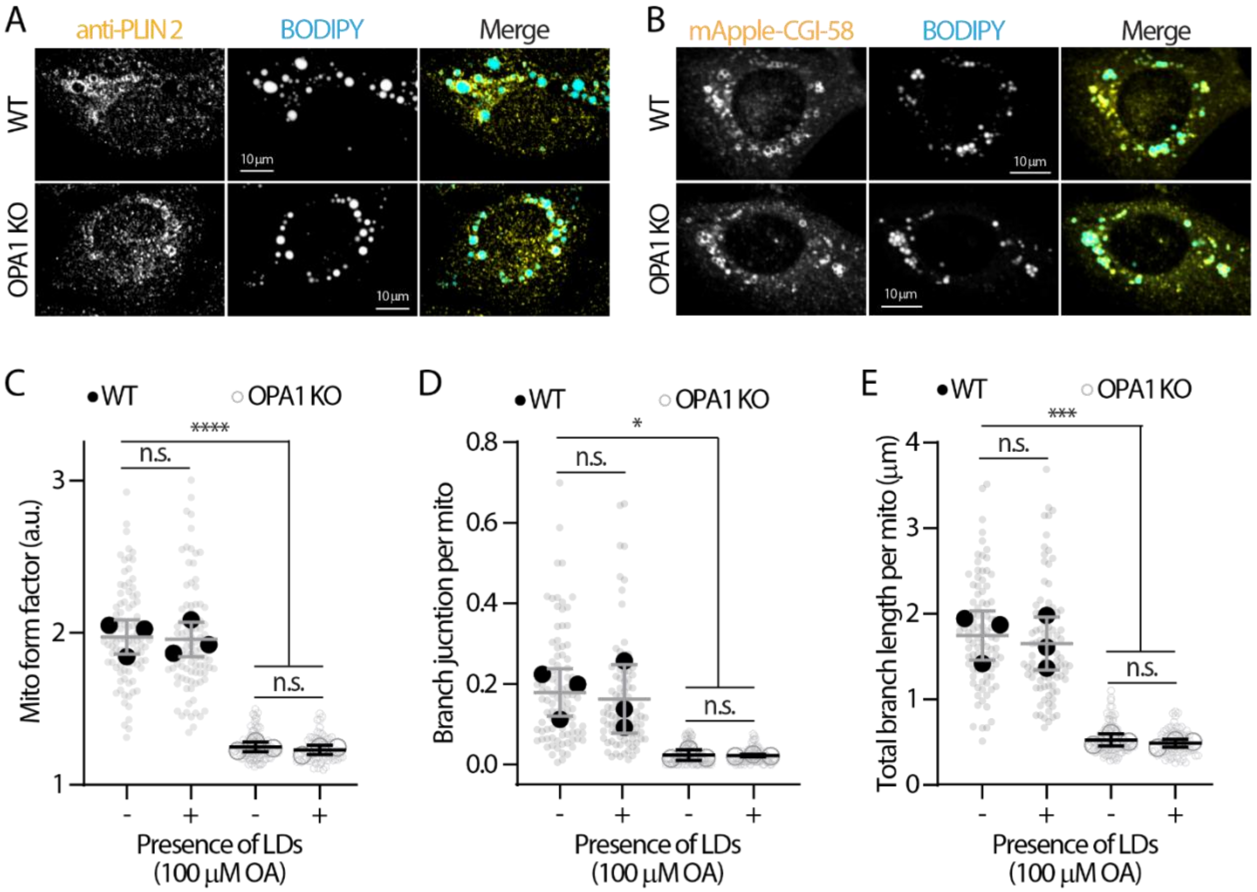

bioRxiv preprint doi: https://doi.org/10.64898/2026.05.07.723579; this version posted May 11, 2026. The copyright holder for this preprint (which was not certified by peer review) is the author/funder, who has granted bioRxiv a license to display the preprint in perpetuity. It is made available under a CC-BY 4.0 International license.

Figure S5. Lipid droplet protein distributions and mitochondrial characteristics in wildtype and *OPA1* knockout U2OS cells. (A) Localization of endogenous PLIN 2 on lipid droplets (LDs) labeled with BODIPY-493/503 in wildtype (WT) and *OPA1* knockout (KO) U2OS cells treated with 100 μM oleic acid (OA) overnight. Maximal intensity projected (MIP) confocal images from four axial slices (~1 μm total thickness) are shown. **(B)** Localization of mApple-CGI-58 and LDs labeled with BODIPY-493/503 in WT and *OPA1* KO U2OS cells treated with 100 μM OA overnight. MIP confocal images from four axial slices (~1 μm total thickness) are shown. **(C–E)** Two-dimensional 'mitochondria analyzer' analysis of mitochondrial morphology and connectivity measuring (C) form factor, (D) total branch length, and (E) branch junction in MitoTracker DeepRed–stained WT and *OPA1* KO U2OS cells -/+ overnight 100 μM OA treatment. Mean ± standard deviation from three independent experiments are shown (total of 74–100 cells). For panels C–E, n.s., no significance, ****$P \leq 0.0001$, ***$P \leq 0.001$, *$P \leq 0.05$, as assessed by one-way ANOVA.

bioRxiv preprint doi: https://doi.org/10.64898/2026.05.07.723579; this version posted May 11, 2026. The copyright holder for this preprint (which was not certified by peer review) is the author/funder, who has granted bioRxiv a license to display the preprint in perpetuity. It is made available under a CC-BY 4.0 International license.

Table S1. Missense polymorphisms within exon 4 of OPA1 from the GTEx database

Details of missense polymorphisms within exon 4 of OPA1 from the GTEx database are indicated, including gnomAD identification number (gnomAD ID), chromosome of this polymorphism (Chromosome), the reference single nucleotide polymorphism (SNP) cluster identification number (rsIDs), the reference and alternate nucleotide of the SNP. The predicated consequence of the SNP (HGVS Consequence), at protein level (Protein Consequence) and at transcript level (Transcript Consequence), the effect of the SNP (VEP Annotation) and the frequency of this SNP, sorted from high to low (Allele Frequency) are also listed.

Table S2. Lipid-related traits analyzed from the SJLIFE database

Association results for lipid-related traits from SJLIFE, including trait name (trait), measurement scale using raw or inverse-rank normal transformation (IRNT) (selected_scale), and source of measurements (selected_column). The statistical model (model) and PLINK test (plink_test) are shown. Effect estimates (effect) with standard error (se), test statistic (stat), p-value (p_value), and sample size (observed count) are reported. Alleles are indicated (ref, alt, effect_allele). Benjamini–Hochberg FDR-adjusted p-values are provided (bh_FDR_primary_set).

Table S3. Oligoes used in this study

Oligo names, sequences (5’-3’) and the fragments used to amplify (Note) are listed.

Table S1 Polymorphism within exon 4 of OPA1

gnomAD ID	rsIDs	Reference	Alternate	HGVS Consequence	Protein Consequence	Transcript Consequence	VEP Annotation	Allele Frequency (high to low)
3-193617202-G-A	rs7624750	G	A	p.Ser158Asn	p.Ser158Asn	c.473G>A	missense_variant	0.47097541
3-193617259-G-T	rs150279202	G	T	p.Ser177Ile	p.Ser177Ile	c.530G>T	missense_variant	0.000106719
3-193617246-G-A	rs778997114	G	A	p.Val173Ile	p.Val173Ile	c.517G>A	missense_variant	6.50941E-05
3-193617234-TTTG-T	rs760263649	TTTG	T	p.Phe169_Asp170delins	p.Phe169_Asp170delins	c.506_508del	inframe_deletion	3.59527E-05
3-193617229-C-T	rs754177232	C	T	p.Pro167Leu	p.Pro167Leu	c.500C>T	missense_variant	1.79778E-05
3-193617184-T-A	rs2108877878	T	A	p.Ile152Asn	p.Ile152Asn	c.455T>A	missense_variant	1.19458E-05
3-193617264-T-A		T	A	p.Leu179Met	p.Leu179Met	c.535T>A	missense_variant	5.58791E-06
3-193617273-T-C	rs769335936	T	C	p.Phe182Leu	p.Phe182Leu	c.544T>C	missense_variant	4.9951E-06
3-193617193-C-T	rs751318725	C	T	p.Ala155Val	p.Ala155Val	c.464C>T	missense_variant	3.12227E-06
3-193617279-A-G		A	G	p.Thr184Ala	p.Thr184Ala	c.550A>G	missense_variant	1.88651E-06
3-193617186-A-G	rs762882041	A	G	p.Arg153Gly	p.Arg153Gly	c.457A>G	missense_variant	1.88238E-06
3-193617260-C-A	rs727504057	C	A	p.Ser177Arg	p.Ser177Arg	c.531C>A	missense_variant	1.86165E-06
3-193617252-A-C	rs1729174080	A	C	p.Ser175Arg	p.Ser175Arg	c.523A>C	missense_variant	1.86017E-06
3-193617235-T-C	rs1386915863	T	C	p.Phe169Ser	p.Phe169Ser	c.506T>C	missense_variant	1.8598E-06
3-193617237-G-A		G	A	p.Asp170Asn	p.Asp170Asn	c.508G>A	missense_variant	1.85974E-06
3-193617221-G-C	rs1380881656	G	C	p.Lys164Asn	p.Lys164Asn	c.492G>C	missense_variant	1.85966E-06
3-193617233-C-G	rs1262531072	C	G	p.Asp168Glu	p.Asp168Glu	c.504C>G	missense_variant	1.85957E-06
3-193617187-G-A	rs766257852	G	A	p.Arg153Lys	p.Arg153Lys	c.458G>A	missense_variant	1.25197E-06
3-193617271-A-G	rs138884045	A	G	p.Asp181Gly	p.Asp181Gly	c.542A>G	missense_variant	1.24537E-06
3-193617269-G-T	rs747354358	G	T	p.Lys180Asn	p.Lys180Asn	c.540G>T	missense_variant	1.24328E-06
3-193617201-A-G		A	G	p.Ser158Gly	p.Ser158Gly	c.472A>G	missense_variant	1.24267E-06
3-193617228-C-T	rs1729170377	C	T	p.Pro167Ser	p.Pro167Ser	c.499C>T	missense_variant	1.23977E-06
3-193617229-C-G	rs754177232	C	G	p.Pro167Arg	p.Pro167Arg	c.500C>G	missense_variant	1.23976E-06
3-193617224-A-C		A	C	p.Leu165Phe	p.Leu165Phe	c.495A>C	missense_variant	1.23968E-06
3-193617181-A-G	rs1329178588	A	G	p.Lys151Arg	p.Lys151Arg	c.452A>G	missense_variant	6.33E-07
3-193617183-A-T		A	T	p.Ile152Phe	p.Ile152Phe	c.454A>T	missense_variant	6.29E-07
3-193617280-C-T		C	T	p.Thr184Ile	p.Thr184Ile	c.551C>T	missense_variant	6.29E-07
3-193617277-T-C	rs772737325	T	C	p.Phe183Ser	p.Phe183Ser	c.548T>C	missense_variant	6.27E-07
3-193617274-T-G		T	G	p.Phe182Cys	p.Phe182Cys	c.545T>G	missense_variant	6.24E-07
3-193617196-T-G		T	G	p.Leu156Arg	p.Leu156Arg	c.467T>G	missense_variant	6.22E-07
3-193617198-C-T		C	T	p.Pro157Ser	p.Pro157Ser	c.469C>T	missense_variant	6.22E-07
3-193617270-G-C	rs780818428	G	C	p.Asp181His	p.Asp181His	c.541G>C	missense_variant	6.22E-07
3-193617268-A-C	rs780451120	A	C	p.Lys180Thr	p.Lys180Thr	c.539A>C	missense_variant	6.21E-07
3-193617266-G-T		G	T	p.Leu179Phe	p.Leu179Phe	c.537G>T	missense_variant	6.21E-07
3-193617263-A-C		A	C	p.Leu178Phe	p.Leu178Phe	c.534A>C	missense_variant	6.21E-07
3-193617207-G-A		G	A	p.Glu160Lys	p.Glu160Lys	c.478G>A	missense_variant	6.20E-07
3-193617254-C-G		C	G	p.Ser175Arg	p.Ser175Arg	c.525C>G	missense_variant	6.20E-07
3-193617255-C-G	rs1729174512	C	G	p.Leu176Val	p.Leu176Val	c.526C>G	missense_variant	6.20E-07
3-193617216-G-A		G	A	p.Val163Ile	p.Val163Ile	c.487G>A	missense_variant	6.20E-07
3-193617244-T-C		T	C	p.Ile172Thr	p.Ile172Thr	c.515T>C	missense_variant	6.20E-07
3-193617235-T-G		T	G	p.Phe169Cys	p.Phe169Cys	c.506T>G	missense_variant	6.20E-07
3-193617238-A-T		A	T	p.Asp170Val	p.Asp170Val	c.509A>T	missense_variant	6.20E-07
3-193617231-G-C		G	C	p.Asp168His	p.Asp168His	c.502G>C	missense_variant	6.20E-07

St. Jude - Confidential

Table S2. Lipids-related traits from SJLIFE database

trait	selected_sca	selected_column	model	plink_test	effect	se	stat	p_value	observed cour	ref	alt	effect_allel	bh_FDR_primary_set
Body fat	raw	TOTAL_PERCENT_FAT	additive	ADD	0.106248	0.169625	0.626367	0.531115	3505	G	A	A	0.652977647
Body fat	raw	TOTAL_PERCENT_FAT	dominant	DOM	-0.183761	0.273774	-0.671214	0.502129	3505	G	A	A	0.652977647
Body fat	raw	TOTAL_PERCENT_FAT	recessive	REC	0.482157	0.280026	1.72183	0.0851883	3505	G	A	A	0.189307333
Body fat	raw	TOTAL_PERCENT_FAT	2df	GENO_2DF			2.35265	0.0952669	3505	G	A	A	0.1905338
Blood TAG	irnt	milli.labs_triglyceride_recent	additive	ADD	-0.0671022	0.020306	-3.30456	0.000959017	4327	G	A	A	0.01918034
Blood TAG	irnt	milli.labs_triglyceride_recent	dominant	DOM	-0.0990229	0.0328879	-3.01092	0.00261963	4327	G	A	A	0.024850867
Blood TAG	irnt	milli.labs_triglyceride_recent	recessive	REC	-0.0794739	0.0334422	-2.37645	0.0175233	4327	G	A	A	0.0700932
Blood TAG	irnt	milli.labs_triglyceride_recent	2df	GENO_2DF			5.59922	0.00372763	4327	G	A	A	0.024850867
Blood cholest	irnt	milli.labs_cholesterol_totl_recent	additive	ADD	-0.0395448	0.0205146	-1.92764	0.0539654	4327	G	A	A	0.154186857
Blood cholest	irnt	milli.labs_cholesterol_totl_recent	dominant	DOM	-0.0843895	0.0332082	-2.54122	0.0110812	4327	G	A	A	0.055406
Blood cholest	irnt	milli.labs_cholesterol_totl_recent	recessive	REC	-0.0199389	0.0337785	-0.590285	0.555031	4327	G	A	A	0.652977647
Blood cholest	irnt	milli.labs_cholesterol_totl_recent	2df	GENO_2DF			3.26451	0.0383096	4327	G	A	A	0.127698667
LDL	irnt	milli.labs_ldl_cholesterol_recent	additive	ADD	-0.0197934	0.0208515	-0.949257	0.342543	4297	G	A	A	0.570905
LDL	irnt	milli.labs_ldl_cholesterol_recent	dominant	DOM	-0.0603581	0.0337426	-1.78878	0.0737212	4297	G	A	A	0.184303
LDL	irnt	milli.labs_ldl_cholesterol_recent	recessive	REC	0.00878604	0.0343235	0.255978	0.79798	4297	G	A	A	0.79798
LDL	irnt	milli.labs_ldl_cholesterol_recent	2df	GENO_2DF			2.00567	0.134696	4297	G	A	A	0.244901818
HDL	irnt	milli.labs_hdl_cholesterol_recent	additive	ADD	0.0143022	0.0207238	0.690136	0.490146	4327	G	A	A	0.652977647
HDL	irnt	milli.labs_hdl_cholesterol_recent	dominant	DOM	0.0283716	0.0335565	0.84549	0.397884	4327	G	A	A	0.612129231
HDL	irnt	milli.labs_hdl_cholesterol_recent	recessive	REC	0.00943222	0.0341111	0.276515	0.782166	4327	G	A	A	0.79798
HDL	irnt	milli.labs_hdl_cholesterol_recent	2df	GENO_2DF			0.357355	0.699545	4327	G	A	A	0.777272222

St. Jude - Confidential

Table S3. Oligoes used in this study

Primers	Oligo sequence (5'-3')	Note
Opa1f-SalI	aaaa GTCGACatgtggcgactacgt	To amplify Opa1 from cDNA
Opa1r-BamHI	aaaa GGATCCCGtttctcctgatgaag	
5'Opa1-158N/S	gaaaagcccttcctaGttcagaagaccttgt	Correct 158N to 158S
3'Opa1-158N/S	acaaggtcttctgaaCtaggaagggcttttc	
5'Opa1-S158N	gaaaagcccttcctaAttcagaagaccttgt	To generate Opa1 S158N
3'Opa1-S158N	acaaggtcttctgaaTtaggaagggcttttc	
YFP Kozak remover f	GATCCACCGCTCGCCGTGAGCAAGGGCGAG	To remove the Kozak sequence in front of YFP in Opa1-YFP
YFP Kozak remover R	CTCGCCCTTGCTCACGGCGAGCGGTGGATC	
5'Yfp-Opa1 ΔMTS	CGAATTCTGCAGTCGACatgttttggccagcaagattagct	To generate Opa1 ΔMTS-YFP
3'Yfp-Opa1 ΔMTS	agctaatcttgctggccaaaacatGTCGACTGCAGAATTCG	
5'Yfp-Opa1 iso2	gatgagtatatcgattttggttctccggaagaaacg	To generate Opa1 isoform 2
3'Yfp-Opa1 iso2	cgtttcttccggagaaccaaaatcgatatactcatc	
A195stop F	gaaacggcgtttagagcaTAacagatcgtggatctgaa	To generate Opa1 truncations ending at aa195
A195stop R	ttcagatccacgatctgtTAtgctctaaacgccgtttc	
5'Yfp-Opa1 127-c	CGAATTCTGCAGTCGACccggaccttagtgaatat	To generate Opa1 truncations starting at aa127
3'Yfp-Opa1 127-c	atattcactaaggtccggGTCGACTGCAGAATTCG	
5'YFP-Opa1 157-c	CGAATTCTGCAGTCGACATGcctaGttcagaagacctt	To generate Opa1 truncations starting at aa157
3'YFP-Opa1 157-c	aaggtcttctgaaCtaggCATGTCGACTGCAGAATTCG	
gfp remover Tre lenti f	CCGGTCGCCACCATGGTGATGTctagactggacaag	To remove GFP from the 5' of YFP on Tre-iOpa1-YFP
gfp remover Tre lenti r	cttgtccagtctagACATCACCATGGTGGCGACCGG	
5'lenti-iOpa1	cgagcgtacgccaccatgtggcgactacgt	To generate TRE-OPA1-YFP-E3G
3'Lenti-iOpa1	TCTTTTATTCTAGCTaAGCTTACTTGTACAG	
5' L156A	aaaattagaaaagccGCtcctaGttcagaagac	To introduce L156A mutation
3' L156A	gtcttctgaaCtaggaGCggcttttctaatttt	
5' F169A	aagttagcaccagacGCtgacaagattgttgaa	To introduce F169A mutation
3' F169A	ttcaacaatcttgtcaGCgtctggtgctaactt	
ATGL f	ATTCCGTACGCCACCatgtttccccgcgag	To amplify ATGL from cDNA
ATGL R	aaaaACCGGTCCACCTCCcagccccagggccccgat	
ATGL S47A f	cacatctacggcgccgcggccggggcgctcacg	To generate ATGL dead mutant S47A
ATGL S47A r	cgtgagcgccccggccgcggcgccgtagatgtg	
CGI58F	aaaa AAGCTTaGCGGCGGAGGAGGAG	To amplify CGI58 from cDNA
CGI58F	aaaa GGATCCTCAGTCCACAGTGTCGCA	
exon4-gBlock	CTGCAGTCGACaccatggagaaaattagaaaagcccttcctaGttcagaagaccttgtaaagt<br>tagcaccagactttgacaagattgttgaaagccttagcttattgaaggactttttttacctcaggtggcggagg<br>gggatcAcGGGATCCACCG	synthetic fragment containing exon 4
mhYFP-gBlock	CTGCAGTCGACaccatggagaaaattagaaaagcccttcctaGttcagaagaccttgtaaagt<br>tagcaccagactttgacaagattgttgaaagccttagcttattgaaggactttttttacctcaggtggcggagg<br>gggatcAcGGGATCCACCG	synthetic fragment containing mhYFP
**RT-PCR primers**		
hOpa1 RT-F	TACGCAAGATCATCTGCCAC	
hOpa1 RT-R	GAACGTGTCATCATCTCCCC	
hOpa1 RT 2-3F	GCTACACAGCCAAAAAGACT	
hOpa1 RT 3-5R	TCCGGAGAACCAAAATCGA	Combined with hOpa1 RT 2-3F to amplify fragments from isoform 2 (and exon 4 lacking isoforms)
hOpa1 RT 4-5R	CCGGAGAACCTGAGGTAAA	Combined with hOpa1 RT 2-3F to amplify fragment from isoform 1 (and exon 4 containing isoforms)
hGAPDH RT-F	GCCATCAATGACCCCTTCAT	
hGAPDH RT-R	GCTCCTGGAAGATGGTGATG	

St. Jude - Confidential
